# Complex effects of climatic variation on bumblebee queen fitness

**DOI:** 10.1111/1365-2656.70140

**Published:** 2025-09-19

**Authors:** C. Ruth Archer, Paul Schmid‐Hempel, Regula Schmid‐Hempel, Lena Wilfert

**Affiliations:** ^1^ Institute for Evolutionary Ecology and Conservation Genomics University of Ulm Ulm Germany; ^2^ Institute of Zoology University of Regensburg Universitätsstraße 31 Regensburg Germany; ^3^ Institute of Integrative Biology (IBZ) ETH Zürich Zürich Switzerland

**Keywords:** bumblebee, climatic change, life‐history, parasites, pollinators

## Abstract

1. Climate change is a global biodiversity threat. To understand how a changing climate affects individual fitness and gain insights into which mechanisms are responsible, we need to establish how climatic variation affects individual life‐history traits (e.g. growth, survival and reproduction). Long‐term data linking insect life histories and climate parameters are therefore valuable but, unfortunately, rare.
2. Here, we test how climatic variation affects health, survival and reproduction in a key European pollinator, the buff‐tailed bumblebee *Bombus terrestris*. We relate climatic variation experienced by developing queens in the field, caught between 2000 and 2014, to fitness traits assayed in these queens under otherwise constant lab conditions.
3. We show that wet years consistently reduce queen fitness, while warm temperatures have positive and negative impacts. Behind these annual effects lies strong seasonality. In particular, climatic conditions experienced by young queens as they forage, mate and enter hibernation are vital determinants of whether they reproduce the following spring.
4. Results suggest that climatic drivers that reduce queen resource acquisition prior to hibernation, or accelerate resource loss over winter, are especially detrimental to spring queen fitness. This suggests a strategy to mitigate the negative effects of climate change on bumblebees: ensuring high‐quality forage late in summer before queens enter diapause.

## Graphical abstract

A dearth of data on the health of wild‐insect populations hinders efforts to fully grasp the extent of historical population declines or unravel the mechanisms driving these effects. We help fill this data deficit by testing how climatic variation affects health, reproduction, size and survival in bumblebees—important insect pollinators.

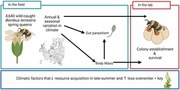

## 1 INTRODUCTION

Many insect species are in decline (Wagner, 2020). These declines are driven by multiple factors (Blüthgen et al., 2023; Wagner, 2020; Wagner et al., 2021), but among these, the threat posed by climate change is global and growing (Blüthgen et al., 2023; Urban, 2015). Identifying which species are most vulnerable to climatic change, and devising strategies to protect those species, requires understanding how species are responding to climatic variation and characterising the mechanisms underpinning these responses (Albaladejo‐Robles et al., 2023). A key part of this puzzle is determining how climate affects individual life‐history traits (e.g. schedules of investment in survival, growth and reproduction) (Paniw et al., 2021). Life‐history responses to variation in climatic conditions have population level impacts, since individual growth, reproduction and survival affect the size and demography of a population (e.g. vital rates) and, therefore, population dynamics (Ehrlén & Morris, 2015). Vice versa, a species' life‐history strategy can influence its vulnerability to climatic change and extinction risk more generally (Albaladejo‐Robles et al., 2023; Hernández‐Yáñez et al., 2022).

Linking climate and life‐history is complex. Effects of climatic drivers may vary in time (e.g. show seasonal effects) (Paniw et al., 2019) or in space (e.g. between populations) (DeMarche et al., 2019) and have contrasting impacts on specific demographic groups (Petry et al., 2016). Furthermore, different climatic variables can interact with one another or with other factors such as food availability or population density (Urban et al., 2016). This complexity means that the impacts of climatic variation on individuals often depend on which population, trait or life‐cycle stage is studied (Paniw et al., 2021). However, for the vast majority of species we lack long‐term, high‐resolution data capable of capturing this complexity (Compagnoni et al., 2021; Paniw et al., 2021). For example, existing data linking climate and life‐history seldom test for interactions between different climatic drivers (Paniw et al., 2021). This data deficit means that our understanding of how climatic variation affects individual life‐history traits or population vital rates (i.e. population measures of growth, survival, reproduction) is incomplete, even in well‐studied taxa like plants and mammals (Compagnoni et al., 2021; Paniw et al., 2021). In insects more generally, there is an urgent need for demographic data (Wagner, 2020), the lack of which makes it hard to relate climate change to individual fitness, life‐history or population trajectories. This is certainly true of wild bees.

Bees pollinate wild flowers (Corbet et al., 1991) and crops (Calderone, 2012; Garibaldi et al., 2011; Klein et al., 2007) and so promote the integrity of semi‐natural habitats (Corbet et al., 1991), food security (Bailes et al., 2015) and public health (Wilfert et al., 2020). However, many wild bee species are declining. This includes bumblebees, where almost one in four species on the IUCN Red List is vulnerable or endangered (Cameron & Sadd, 2020). Bumblebee losses are driven by multiple interacting factors (Cameron & Sadd, 2020; Wilfert et al., 2020), including climate change. Characteristically, and with few exceptions, bumblebees are adapted to cool or cold climates (Woodard, 2017). When temperatures exceed species' thermal limits, bumblebee patch occupancy declines (Soroye et al., 2020) and bumblebees do not appear to move their ranges to mitigate climate change effects (Kerr et al., 2015). This leads to predictions of pronounced range reductions under global warming scenarios (Martínez‐López et al., 2021). Conversely, moderate warming may have positive impacts on bees across parts of their range (Soroye et al., 2020), for example, by improving flight performance, which is generally poor at low temperatures (Kenna et al., 2021). Altered patterns of precipitation can also lead to local bumblebee extinctions (Soroye et al., 2020). The exact reasons why variation in climatic drivers is having such pronounced impacts on bumblebees are not yet fully understood (Johnson et al., 2023). Winter warming may elevate the metabolic costs of diapause (CaraDonna et al., 2018), while high summer temperatures could cause heat injury to workers or larvae, and this reduces colony reproduction (reviewed in White & Dillon, 2023). Climate change could have costly impacts on bees by reducing forage availability (Miller‐Struttmann et al., 2015) or by favouring the spread of pathogens or parasites (Johnson et al., 2023). Fine‐scale data linking climatic variation and bumblebee health and life history using wild‐caught animals could further elucidate how climate affects bees and reveal the underlying mechanisms. This, in turn, may help us devise conservation strategies to mitigate any negative impacts of a changing climate on bees and the vital pollination services that they provide.

**FIGURE 1:**
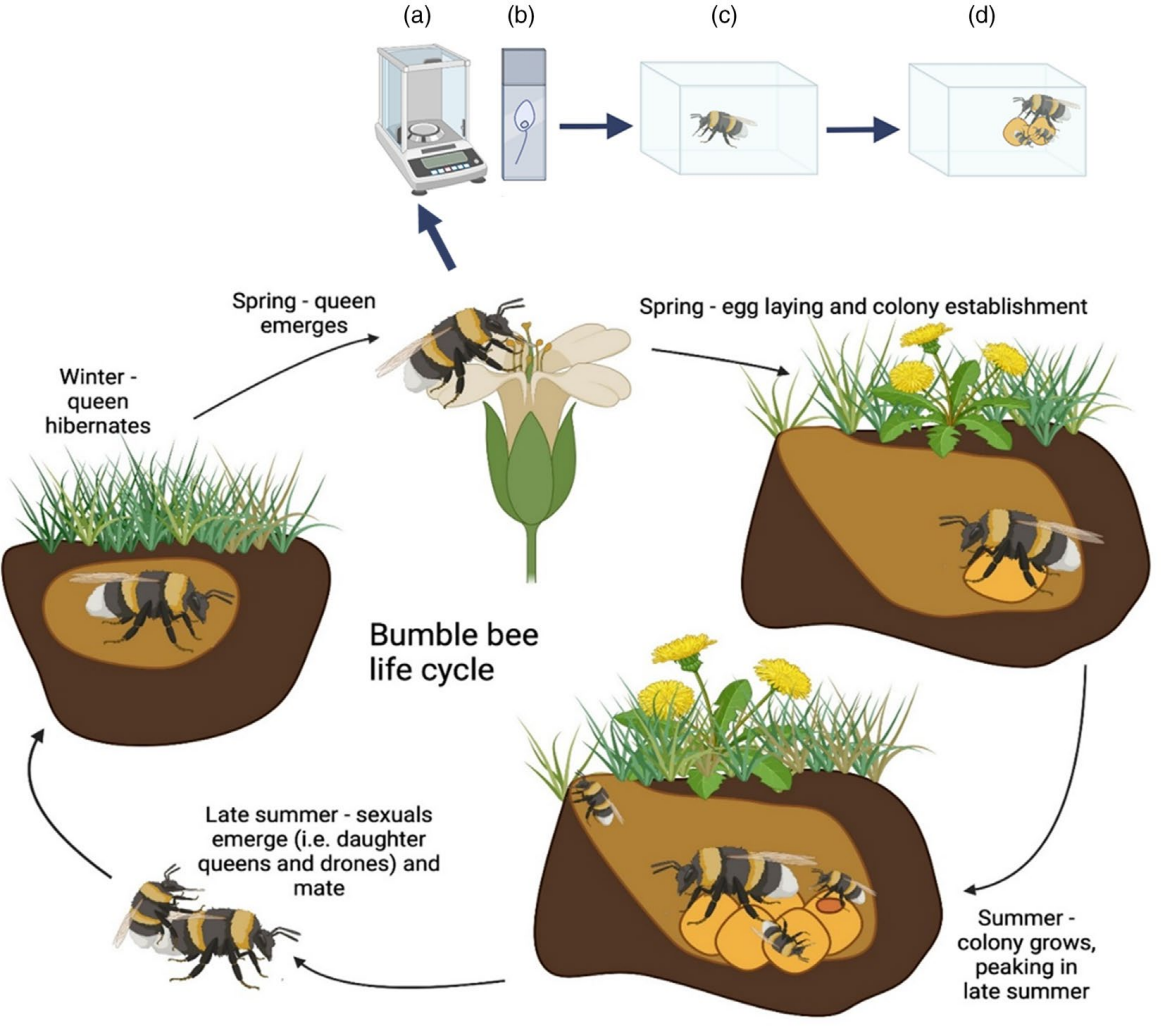
The annual life cycle of *Bombus terrestris* and summary of traits measured in the lab (a–d). Life cycle: Queens emerge from hibernation in the spring, when they consume nectar and pollen and search for a nest site. On finding a site, queens lay eggs that give rise to non‐reproducing daughters (i.e. workers), who forage and help the queen establish and expand the colony. From this point on, queens remain in the colony and lay eggs that give rise to more workers until summer, when sexuals (i.e. daughter queens and drones) are produced. Sexuals then leave the colony to mate. Only the fertilised daughter queens survive and enter diapause, hibernating in the ground until spring when this cycle restarts with the surviving queens emerging from hibernation. Data collection: Queens were collected in spring when they emerged from hibernation. The following traits were then assayed: (a) body mass—a marker of overall condition, (b) *Crithidia* infection—a gut parasite that reduces *B. terrestris* queen fecundity (Yourth et al., 2008), (c) survival after emergence from hibernation and (d) worker production (a key aspect of reproductive success and so from hereon, reproduction). Mass and parasite infection were assayed immediately after catching queens but survival and reproduction were measured in queens held under standardised laboratory conditions. Figure created by CRA in BioRender.

We use data from wild‐caught *Bombus terrestris* queen bees collected in each of 13 years from two sites in northern Switzerland (Figure S1; Table S1) to explore how climatic variation (the difference between temperature and precipitation that bees experienced in the field, relative to historic conditions) is related to four measures of bee fitness: weight, parasite infection status, reproduction and survival. We use mixed models to test for relationships at an annual scale, and sliding window analyses to explore seasonal effects. While *B. terrestris* is a social insect with an annual life cycle (Figure 1), we focus on queens which spend much of their lives in a solitary state and are therefore particularly vulnerable to climatic variation (Woodard, 2017). Moreover, queens are the reproductive caste, responsible for producing the daughter queens and drones that, ultimately, signal colony success. We relate variation in temperature and precipitation to queen survival (days post capture), reproduction (a binary measure of whether queens established colonies or not, as demonstrated by the production of workers), health (a binary measure of whether queens were infected with the gut parasite *Crithidia* or not) and a marker of overall condition (body mass). Collectively, this enables us to determine whether the same climatic driver has opposing fitness impacts via different traits, and explore interactive effects between climatic drivers—both of which are poorly understood (Paniw et al., 2021). By linking queen survival and reproduction with a trait that indicates condition (body mass) we can determine if impacts of climate differ between high and low quality individuals. Finally, by characterising climate impacts on *Crithidia*, we shed light on how a widespread bee parasite is responding to climate change alongside its host. This builds a detailed understanding of how climatic variation affects the health and fitness of wild‐caught queen bumblebees and in doing so, fills key knowledge gaps that limit our understanding of how a changing climate can affect wild pollinators.

## 2 MATERIALS AND METHODS

### 2.1 Data collection

The queens studied here were caught for the purposes of establishing laboratory colonies for experiments by the Schmid‐Hempel research group at ETH Zurich, Switzerland. Queens were caught and allowed to establish colonies under standardised conditions, which were then used for experiments in summer (usually beginning in mid‐June). Queens that had not reproduced by this time were sacrificed by freezing. In this way, 4640 spring queens were caught between 2000 and 2014 from two sites in northern Switzerland (Aesch and Neunforn, for map and geographic co‐ordinates, see Figure S1; Table S1). However, because some of these queens were missing observations, not all queens were used for analyses presented here. For example, queens from the years 2012 and 2013 lacked body mass data and consequently were excluded from further analyses. The number of complete cases used to analyse each variable of interest is shown in Table S2.

Every spring, starting 24–72 h after daytime temperatures exceeded 12° for at least 48 h, queens were collected from each site (Figure S2). The few queens caught carrying pollen were released because these were likely to have established colonies. The species *Bombus lucorum* has been documented in the surveyed regions. However, *B. lucorum* queens can reliably be differentiated from *B. terrestris* queens in our study region through visual observation. Subsequent lab analyses in a subset of focal queens verified the accuracy of these identifications, revealing that cases of misidentification were very rare, with an estimated error rate of approximately 1%. In the lab, queens were weighed, and their faeces microscopically screened for *Crithidia* presence/absence. Queens were established in acrylic glass cages (12.5 × 7.5 × 5.5 cm) with ad libitum pollen and sugar water and then monitored every 2–3 days for egg laying, worker production (our measure of reproductive success), survival and cage conditions. Neither permits for sampling nor ethics approval were needed for this work.

### 2.2 Weather stations and climatic variables

Weather data were collected from the Swiss Federal Office of Meteorology and Climatology (Source: MeteoSchweiz) portal on the 31st of January 2024. Data for the Aesch study site were collected from the nearby Basel/Binningen Weather station (MeteoSchweiz station abbreviation—BAS). For the Neunforn site, precipitation data were available from the nearest weather station (Niederneunforn—NIE) but as this station did not collect temperature data, these data were sourced from the nearby Aadorf/Tänikon station (TAE) (Figure S1; Table S1). All climatic variables analysed were standardized relative to historic norms by MeteoSchweiz (Figures S3–S5). This means that climatic variables reflect deviation between the actual climatic conditions in a particular time period (e.g. a day, week or year) and the average climatic conditions experienced at the same site and over the same time period. These climatic averages are calculated over a 30‐year reference period (here 1991–2020). These standardized variables should be robust to minor variation in absolute temperature or precipitation between each collection site and weather station.

Effects of climate were analysed at two temporal scales. First, spring queen phenotype was related to average measures of temperature and precipitation in the preceding calendar year (i.e. the year the queen was born, developed and entered hibernation). Second, to explore seasonal effects, a sliding window approach was used to relate queen traits to weekly (temperature) and monthly (precipitation) climatic variation. Here, we studied a 12‐month period before the 1st of March in the year each queen was collected. This period spans the time when the queens in our dataset began emerging from hibernation and reaches back to when their own mothers exited diapause.

Accordingly, the following climatic variables were used. The abbreviations used by MeteoSchweiz and the units associated with each variable are shown in brackets:

(1) *Annual air temperature 2 m above ground; deviation of the annual mean from the historic norm* (tre200yv—°C).

(2) *Annual precipitation; annual total in relation to the historic norm* (rre150yv—percentage). Precipitation measures are collected automatically and collection machines are heated, such that snow is included. Annual precipitation and temperature measures were not closely correlated (Figure S5).

(3) *Daily air temperature 2 m above ground; deviation of the daily mean from the historic norm* (tre200dv—°C), aggregated to a weekly level for sliding window analyses.

(4) *Monthly precipitation; deviation of the monthly total from the historic norm* (rre150mv—percentage). Monthly precipitation was the highest resolution available.

### 2.3 Statistical analyses

Analyses were conducted in R version 4.0.5 (R Core Development Team, 2020) using the following packages: *dplyr* (Wickham et al., 2021) for data wrangling, *lme4* (Bates et al., 2015), *car* (Fox & Weisberg, 2019), *DHARMa* (Hartig, 2021), survival (Therneau, 2021), *climwin* (Bailey & van de Pol, 2016) and *piecewiseSEM* (Lefcheck, 2016) for analyses and model checking and *gridExtr*a (Auguie & Antonov, 2017) and *ggplot2* (Wickham, 2016) for plotting.

Data were analysed in three steps:

1. Individual mixed models related each trait to annual climatic measures in the preceding calendar year.
2. Piecewise structural equation modelling (SEM) was used to test for complex relationships between multiple interrelated variables and annual climatic variation.
3. A sliding window approach related to weekly (temperature) or monthly (precipitation) data in the 12 months before queen collection (1st of March–1st of March) to queen phenotype.

This approach balances the risks of multiple testing (i.e. studying traits collected in the same animals in separate models) against the loss of power due to a reduction in sample size if only complete observations are analysed in a single piecewise SEM (Table S2).

#### 2.3.1 Step 1: Spring queen phenotype and annual climatic variation

Individual mixed models were constructed to relate annual climatic variation to mass (continuous variable), reproduction (no workers—0, workers produced—1), survival (days post collection, continuous) and *Crithidia* infection (0/1). Study site (factor, two levels) and collection day of the year (continuous) were included as covariates in all models, as was body mass (continuous), except when analysing effects of climate on body mass itself. In each model, linear effects of temperature and precipitation were included, as was the interaction between them. Year (factor) was modelled as a random intercept. Continuous variables (mass, collection date, temperature and precipitation) were standardised using the R scale function to facilitate model convergence. The general model structure was: Response variable~site + collection day of the year (scaled) + mass (scaled) + temperature (scaled) + precipitation (scaled) + temperature (scaled): precipitation (scaled) + (1|year). For analyses of *Crithidia* and reproduction, binomial generalised linear mixed models were used with the *bobyqa* optimiser to facilitate convergence. For body mass, a linear mixed model was used with a Gaussian error structure. In both these mixed models, *DHARMa* was used to check model fit and for binomial data, fit was assessed using a ‘grouping’ method, which turns 0/1 Bernoulli data into a k/n response. Survival was analysed using mixed cox‐proportional hazards models and deviation from the proportionality assumption was tested for using the function *cox.zph* (for important detail about censoring survival data please see Text S1). The ANOVA function from the *car* package was used to test significance in the full models.

#### 2.3.2 Step 2: Piecewise SEM

The R function *piecewiseSEM* was used to evaluate causal relationships between climatic variables, body mass, *Crithidia* infection status and reproduction. Survival was not included because cox‐proportional hazard models are not supported by the package (Lefcheck, 2016). The starting *piecewiseSEM* model that demonstrates all the hypotheses tested is shown in Figure S6. To facilitate model convergence, climatic variables and collection date were scaled as described above; however, body mass was not because body mass is used as a response variable in this model, as well as an explanatory variable (see Figure S6). In all cases, year was included as a random factor. After creating this starting model, we used *d*‐separation tests to determine if any paths that were not specified in the starting model were significant and Fisher's *c*‐tests to evaluate model fit. We report conditional (*R* ^2^_*c*_) coefficients for each of the models incorporated into the overall SEM, which consider variance associated with fixed and random effects, as well as marginal (*R* ^2^_*m*_) coefficients, which consider variance associated with fixed effects.

#### 2.3.3 Step 3: Sliding window analyses

Sliding window analyses were performed using the *climwin* package, to identify periods of time when the effects of climate on each trait were most pronounced. This approach links a trait and climate in a specific time‐step (e.g. daily) within a defined window (e.g. a year) proceeding a particular date. Every possible window of time in that defined period is modelled (i.e. the window of time modelled slides backwards from the defined date, such that windows vary in start date, end date and thus, duration—for illustration please see Figure S8). This creates multiple models. Next, the best fitting model(s) from the entire collection are identified and the probability of that model being the best model from the broader set is calculated. If more than one model has a high probability of being the best model in the set, model averaging can be used to calculate the average relationship between climate and trait from this collection of high performing models, weighted by the probability that each model in the set is the best model. Finally, because this procedure involves multiple testing, randomisation tests are used to evaluate if there is a significant likelihood of obtaining the level of model support observed for the best model by chance.

Here, we set the 1st of March in the year each queen was collected as a reference date and relate climate in the 12 months preceding this reference date to traits of interest. Our baseline model included the collection day of the year (continuous, scaled) and body mass (continuous, scaled; except where body mass was the response trait). Year (factor) could only be included as a random effect in analyses of continuous traits (i.e. body mass) and for all other models (survival, reproduction, *Crithidia*), year was included as a main effect due to model convergence problems (Table S3). We tested for linear and quadratic climatic effects on each trait, and site was accounted for using the ‘spatial’ term. Queen phenotype data from 2000 were excluded for temperature due to the appropriate climatic variable not being available for both sites in 1999. We report *p* values calculated using 500 randomisations and degrees of freedom equivalent to the number of years multiplied by the number of study sites. When multiple models provided a strong fit to the data, we focus our discussion on the results from model averaging across this set of high‐performing models. Finally, for survival, the top‐performing models in the confidence set suggested multiple climatic peaks. In this case, we split the year into three periods, each encompassing a candidate peak, and repeated the analyses described above for each time period.

## 3 RESULTS

Overall, results show that wet years consistently reduced queen fitness, while warm temperatures had variable impacts, both enhancing and reducing fitness; moreover, these impacts were mediated by different traits. These annual climatic effects overlay strong seasonality, with the direction and magnitude of climatic effects differing between climatic drivers and traits of interest. A common theme, however, is that climatic conditions experienced by young queens as they forage, mate and enter hibernation are vital in determining whether they reproduce the following spring.

### 3.1 Body mass

**FIGURE 2:**
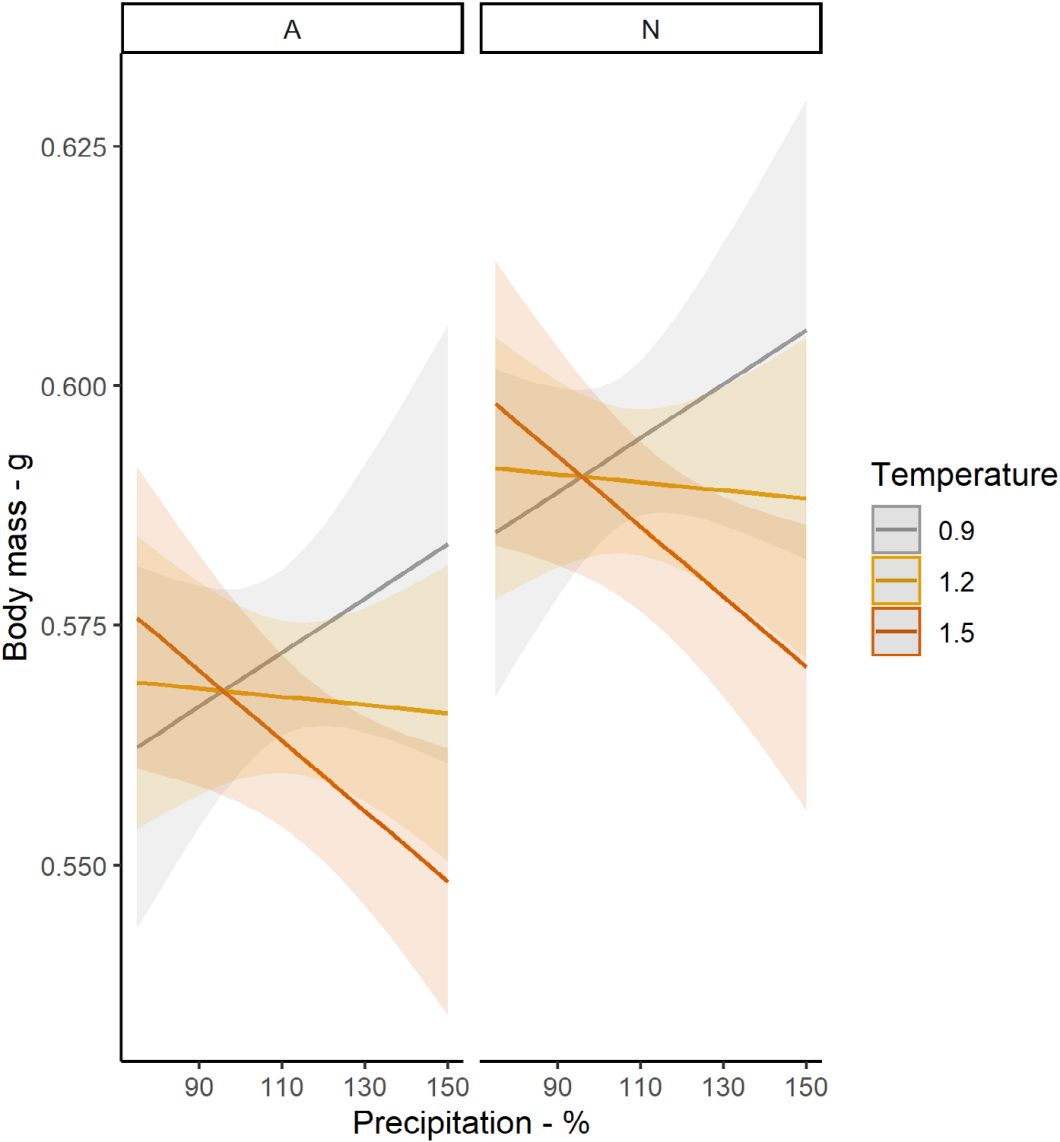
The predicted interaction effect between temperature and precipitation in the preceding year on body mass in Aesch (A) and Neunforn (N). Shaded regions show confidence intervals, and lines show predictions from the model. To plot the interaction, the relationship between precipitation and body mass is shown at the lower, middle and upper terciles of temperature variation—0.9°C (grey), 1.2 (yellow) and 1.5 (orange), respectively. Precipitation is given as percentage deviation relative to the historic norm.

**FIGURE 3:**
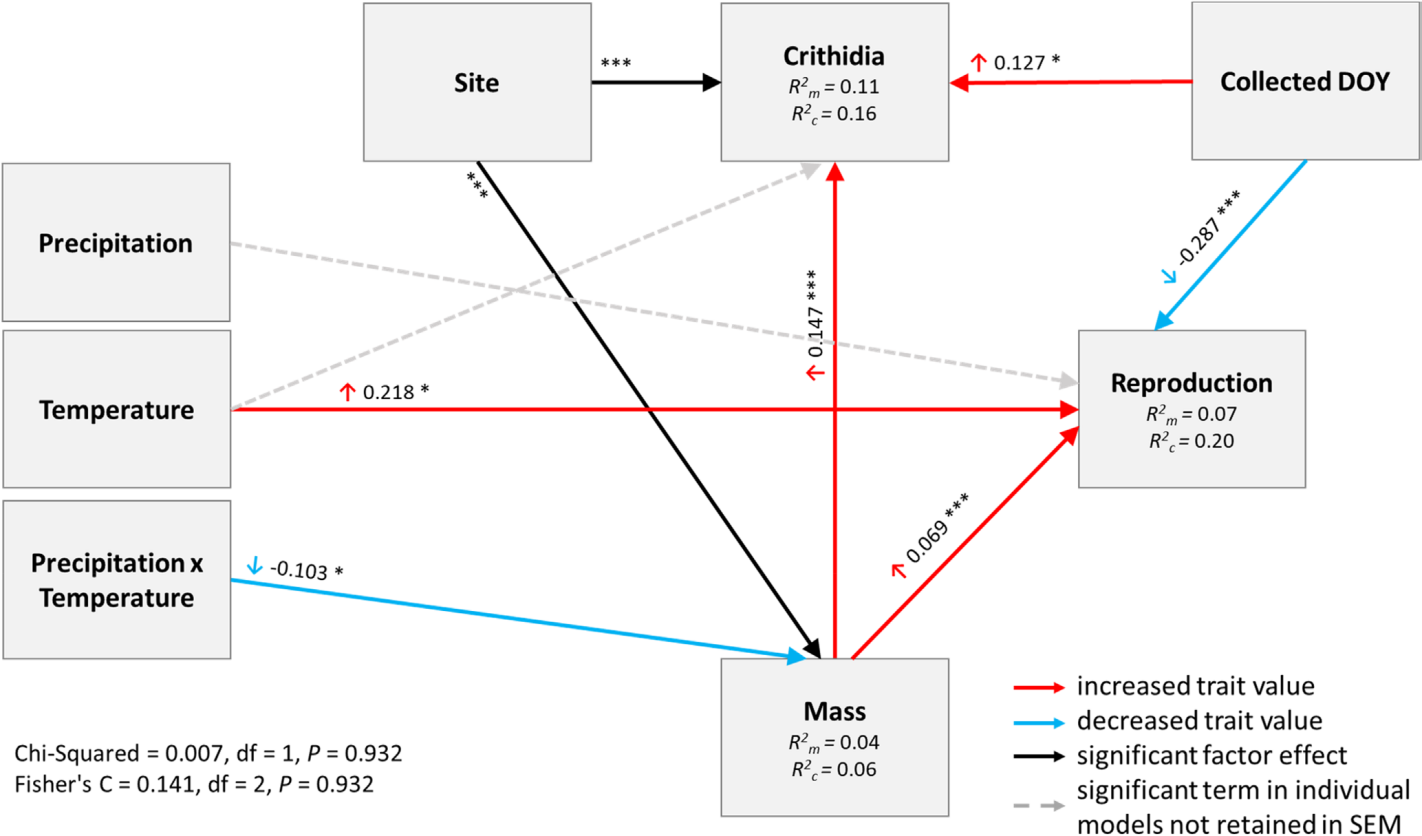
Piecewise Structural Equation Modelling (SEM). Solid arrows show significant effects, with red arrows showing positive and blue arrows showing negative relationships. Black arrows are used for site effects. Grey dashed arrows highlight terms that were significant in the annual models but not in the piecewise SEM approach. Standardised path coefficients are reported next to each of the arrows. *R* ^2^ values (conditional *R* ^2^ *c* and marginal *R* ^2^ *m*) are reported within the boxes for each of the continuous response variables. Stars reflect significance and *p ≤ 0.05; **p ≤ 0.01; ***p ≤ 0.001.

**FIGURE 4:**
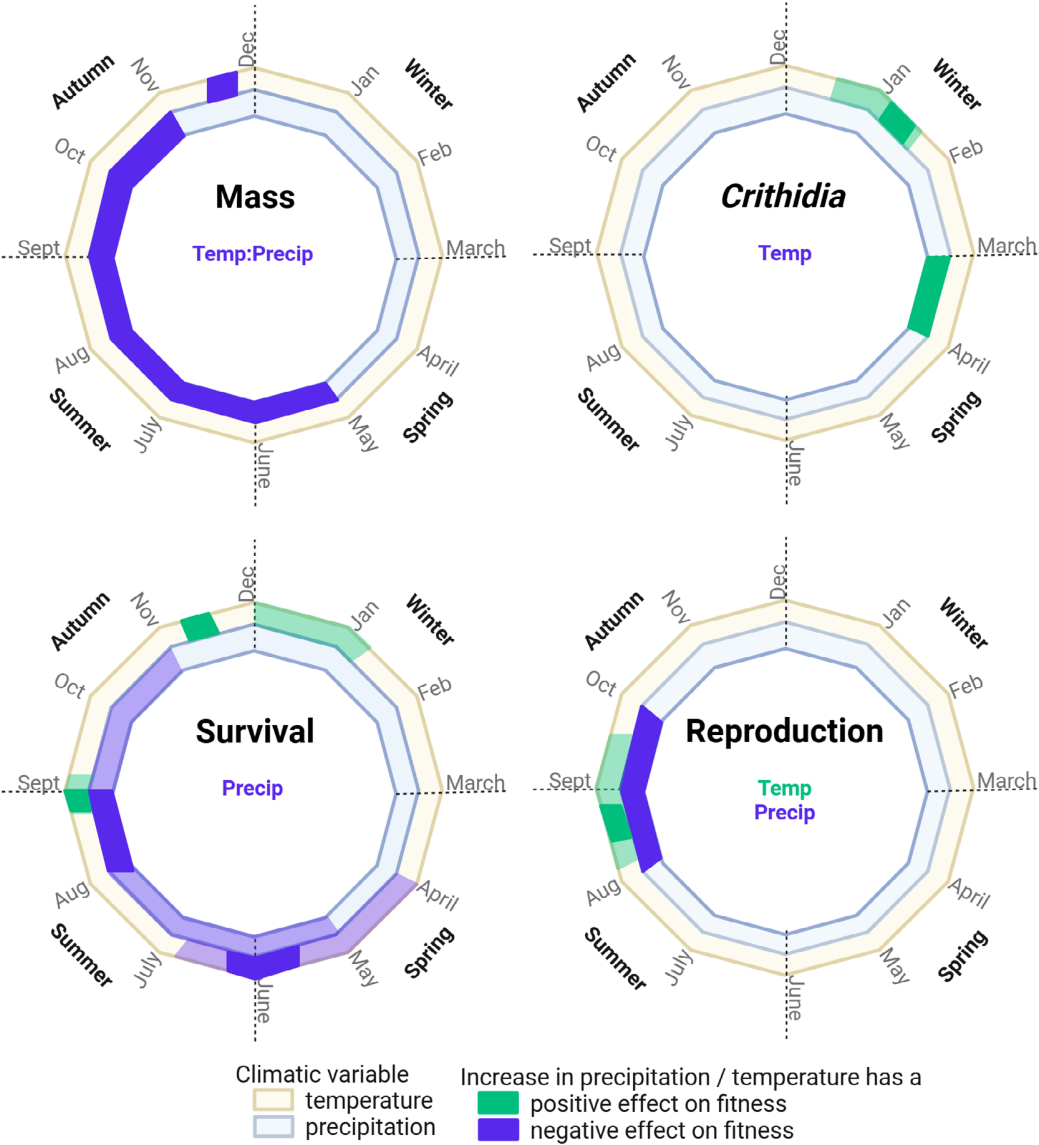
Schematic summarising sliding window analyses. Each ring is divided into monthly steps to represent the year. The outer yellow ring displays results for temperature (weekly steps) and the inner blue ring shows precipitation results (monthly steps). The writing inside each circle summarises the results for annual analyses—only significant climatic effects are shown. Colours show the effect of a rise in each climatic driver on fitness; green means a positive change in fitness and purple a negative change. Light colours indicate windows identified in the confidence set, and dark colours show windows identified in the single best model. Note that the direction of the effect on fitness may oppose the effect direction on the trait, that is survival analyses models the risk of dying and an increased hazard is detrimental to fitness. This is a schematic only and precise estimates of window duration are in Tables S4 and S5.

Collection day did not impact body mass (*χ* ^2^ = 2.26, df = 1, *p* = 0.133) but site did: queens collected from Neunforn were 4.1% heavier than queens collected from Aesch (*χ* ^2^ = 62.78, df = 1, *p* < 0.001). Temperature and precipitation interacted to affect body mass (*χ* ^2^ = 7.26, df = 1, *p* = 0.007; Figure 2): when temperatures were relatively low, precipitation had a positive effect on body mass but the effects of precipitation became increasingly negative with increasing temperature. As a result, warm, wet years correlate with lower body mass. Outputs of piecewise SEM matched these annual modelling results (Figure 3). While global fit statistics suggested that the piecewise SEM fit the data, it is important to note that this model explained little variation in body mass (conditional *R* ^2^, 0.06). At a finer temporal resolution, moving window analyses highlighted that elevated precipitation between May and November, and a warm period in November reduced body mass (Figure 4; Figure S9; Tables S4 and S5).

### 3.2 Crithidia presence/absence

7% of queens from Neunforn were infected with *Crithidia* but 13% of Aesch queens were infected—a significant difference (*χ* ^2^ = 43.10, df = 1, *p* ≤ 0.001). The prevalence of infection was higher in heavier queens (*χ* ^2^ = 18.01, df = 1, *p* ≤ 0.001), and in queens collected later in the year (*χ* ^2^ = 5.52, df = 1, *p* = 0.019). Precipitation did not affect *Crithidia* prevalence singly (*χ* ^2^ = 2.17, df = 1, *p* = 0.141) or via its interaction with temperature (*χ* ^2^ = 0.12, df = 1, *p* = 0.733). However, queens collected after an above‐average warm year were more likely to be infected (*χ* ^2^ = 5.50, df = 1, *p* = 0.019; Figure S7). While effects of site, body mass and collection date were conserved between analytical approaches, the piecewise SEM analysis suggested that climate did not directly affect *Crithidia* incidence (Figure 3). Instead, effects of climate on *Crithidia* were mediated via body mass.

Sliding window analyses suggested significant impacts of temperature and precipitation on *Crithidia* incidence (Figure 4; Figure S10; Tables S4 and S5). The best fitting model suggested that the likelihood of *Crithidia* infection was reduced by warm temperatures in mid‐January. Similarly, a reduction in *Crithidia* prevalence was suggested by averaging models in the confidence set, in particular, by warm temperatures from late in December to mid‐January. Note that these analyses suggest that warm winters reduce infection but overall warm years (see above) increase infection. Analyses of precipitation data suggested that wet conditions in March in the year preceding spring queen emergence (when the mothers of focal queens were emerging and establishing their colonies) reduced *Crithidia* incidence.

### 3.3 Survival

On average, 17% of queens died naturally before the end of the observation period. The risk of dying was lower in heavier queens (hazard ratio coefficient ± standard error: −0.12 ± 0.05, ANOVA: *χ* ^2^ = 7.52, df = 1, *p* = 0.006). Queens collected later in the season (coefficient ± standard error: 0.31 ± 0.09, ANOVA: *χ* ^2^ = 11.80, df = 1, *p* ≤ 0.001) and after wet (preceding) years (coefficient ± standard error: 0.19 ± 0.12, ANOVA: *χ* ^2^ = 10.95, df = 1, *p* = 0.001) had a higher risk of dying. Neither site (*χ* ^2^ = 2.01, df = 1, *p* = 0.156) nor temperature (*χ* ^2^ = 2.69, df = 1, *p* = 0.101) significantly affected this risk. The interaction between climatic variables did not affect hazard (*χ* ^2^ = 0.60, df = 1, *p* = 0.437).

The single best model produced by sliding window analyses suggested that high levels of precipitation in August relative to historic norms increased mortality risk. The broader confidence set suggested that precipitation between May and October was particularly detrimental (Figure 4; Figure S11; Tables S4 and S5). Seasonal effects of temperature were complex. The first model relating temperature across the entire year to mortality risk suggested three influential climatic windows (Figure S11). Thus, further models were run for periods corresponding to each peak (0–19 weeks prior to the 1st of March; 20–29 and 30–52 weeks). The first period focussed on temperatures between mid‐October and March 1st in the year that spring queens were collected. This analysis suggests that warm conditions spanning November, December and January decreased the risk of dying (Table S5). The single best model for the second period (i.e. a second step back in time, 20–29 weeks prior to the 1st of March) suggested that warm periods spanning late August to the start of September reduced the likelihood of spring queens dying prior to reproducing the following spring. The broader model set encompassed this single best model but suggested that the influential window of time was broader, reaching into September (Table S5). Analyses of the third time period (30–52 weeks prior to the 1st of March) suggested that high temperatures in mid‐May to early June elevated mortality risk. The confidence set suggested that high temperatures from April until mid‐June significantly elevated mortality risk (Table S5).

### 3.4 Reproduction

**FIGURE 5:**
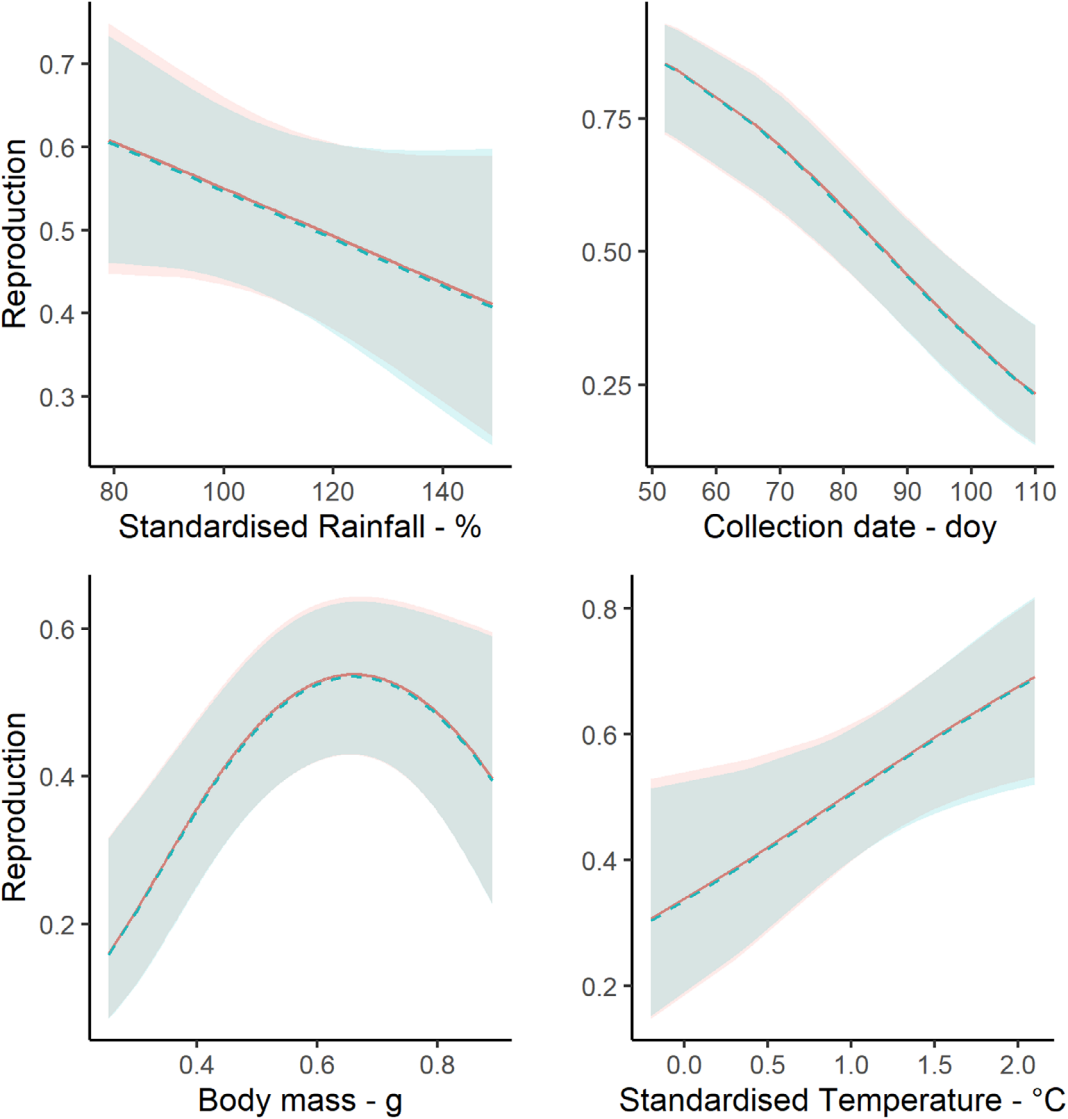
The predicted relationship between the likelihood of successful reproduction and queen body mass (g), the day of the year when queens were collected, standardised rainfall (%—linear) and temperature (°C—linear). Shaded regions show confidence intervals, and lines predictions from the model estimated using the *ggpredict* function. Red, site Aesch; Blue, site Neunforn.

49.5% of queens analysed produced workers, although there was pronounced variation across years (e.g. 35%–82%). The fit of the reproduction model was improved by including a quadratic effect of mass (*χ* ^2^ = 9.25, df = 1, *p* = 0.002). This means that while heavier queens were more likely to reproduce (i.e. positive linear effect of mass, *χ* ^2^ = 11.70, df = 1, *p* = 0.001), the benefits of being large had diminishing returns. Reproduction was independent of site (*χ* ^2^ = 0.02, df = 1, *p* = 0.892) but greater in queens collected earlier in the season (*χ* ^2^ = 25.98, df = 1, *p* ≤ 0.001). Temperature (*χ* ^2^ = 6.65, df = 1, *p* = 0.010) and precipitation in the preceding year (*χ* ^2^ = 5.41, df = 1, *p* = 0.020) affected queen reproduction; warm preceding years increased the likelihood of reproduction, while wet years reduced it (Figure 5). Interactions between climatic variables did not significantly affect reproduction (*χ* ^2^ = 0.50, df = 1, *p* = 0.482). In the piecewise SEM analyses, no significant direct effect of precipitation on reproduction was found. However, precipitation affected body mass via an interaction with temperature, which in turn affected reproduction (Figure 3). That is, the effect of precipitation on reproduction was indirect and mediated by impacts on body mass. Moving window analyses suggested that warm conditions spanning August and September were particularly influential and increased the likelihood of reproduction. Pronounced precipitation in August and September appeared to reduce subsequent reproduction (Figure 4; Figure S12; Tables S4 and S5).

## 4 DISCUSSION

*Bombus terrestris* is an important pollinator (Velthuis & van Doorn, 2006) and climatic effects on its survival and reproduction could have impacts extending far beyond the health of this single species, for example disrupted pollination services and food security. Understanding the mechanisms underpinning any deleterious effects of climatic variation in this species is a critical step towards mitigating them (Johnson et al., 2023). We show that queens emerging after particularly wet years experienced general reductions in fitness but that the fitness consequences of developing in warm years were mixed. These annual climatic effects were largely mediated via body mass, and relationships between climate and phenotype at an annual scale masked seasonal variation in climate effects. Despite this complexity, a picture emerges whereby queens appear most vulnerable to climatic variation late in summer, when they forage, mate and enter hibernation, and during hibernation itself. This suggests that climatic conditions that reduce resource acquisition in summer, or exacerbate resource depletion overwinter, are vital determinants of spring queen fitness.

Wet years reduced spring queen fitness: queens emerging after a wet year were less likely to survive and reproduce, while emerging after warm–wet years reduced spring queen body mass. Queens emerging after warm years were also more likely to host the gut parasite *Crithidia*. Emerging after warm years had no impact on survival and even improved queen reproduction. However, analysing the effects of annual climatic variation on each fitness trait separately presents an incomplete (and somewhat misleading) picture. This is because a SEM suggests that the effects of climatic variation on reproduction and *Crithidia* infection are partly mediated by climate impacts on body mass. The SEM showed that while warm conditions directly improved queen reproduction, warm–wet years reduced body mass and lighter bees were less likely to establish colonies. That is, warm–wet years had an indirect negative impact on reproduction mediated by body mass, that somewhat offset the positive direct effect of warming. Similarly, in this SEM neither climatic factor directly affected *Crithidia* infection, but the temperature–precipitation interaction reduced body mass, and lighter bees were less likely to host *Crithidia*. Evidently, at an annual scale, the interaction between precipitation and temperature is an important determinant of queen fitness, largely due to impacts on body mass.

That warm years contribute towards reduced body mass is not surprising; body mass declines as temperatures rise in many species (Daufresne et al., 2009). This effect is often attributed to warming reducing food availability or quality (Gardner et al., 2011). Some climatic impacts we observe are probably associated with resource availability. For example, wet conditions spanning spring, summer and autumn reduced body mass and spring queen survival, which might reflect that rainfall restricts foraging activities and high humidity reduces pollen retrieval (Karbassioon et al., 2023; Reeves et al., 2024). In contrast, warm and dry conditions in August and September improved queen survival and reproduction. Warm, dry conditions late in summer may improve forage availability, at a time when in some landscapes there is a nectar deficit for bumblebees (Timberlake et al., 2019). Warm, dry conditions could also improve how readily foragers collect these resources late in the season because low temperatures restrict forager flight (Kenna et al., 2021). By affecting forage availability or foraging efficiency, warm‐dry periods late in summer could increase queen resource stores before hibernation, which in turn improves their survival and reproduction in spring. In concert with positive impacts of warm conditions late in summer, we hypothesise that warming reduces queen body mass by increasing resource expenditure overwinter.

The high costs of overwintering—and the potential of climate to mediate these costs—are clear in our results. Strikingly, warm winters reduced spring queen body mass. This likely illustrates that warm winters disturb queens from their quiescent state and in doing so, elevate their metabolic rate. This accelerates how rapidly queens utilise their lipid stores and reduces the resources available to invest in reproduction when they emerge in spring (Ghosh et al., 2017; Hahn & Denlinger, 2007). Similar patterns are seen in *Osmia ribifloris*, an early‐season solitary bee, where experimental warming overwinter reduced body mass (CaraDonna et al., 2018). In contrast, large *B. lucorum* queens emerging after induced diapause have more body fat if they overwintered at warm temperatures, but this trend is reversed in small queens—that is, the direction of effect depends on queen condition (Vesterlund et al., 2014).

Climatic conditions that increase diapause duration may also be detrimental to queen fitness. As discussed above, warm and dry conditions in August and September improved queen survival and reproduction in spring. In mountainous populations in south‐eastern Germany, queens are rarely observed foraging (and thus may have begun hibernating) after July (see data from Sponsler et al., 2022). In other lowland populations in southern Germany and the UK, new queens and drones are often on the wing later in summer (Frank, 1941; Haas, 1946) (pers. comm. Richard Comont [UK BeeWalk] and Hannah Burger [Germany, BienABest]). This means that in late summer, many queens in our Swiss populations will have entered hibernation. Warming in this critical window may delay the onset of hibernation, reduce its length and thus, metabolic costs. This may also explain why being collected later in spring was associated with reduced survival and reproduction; these queens have remained in hibernation longer, further depleting the resources needed to successfully establish colonies. In sum, climatic factors that elevate the costs of overwintering may have negative impacts on queen fitness.

Given these negative impacts of warm winters, why do warm winters also reduce spring queen mortality risk and *Crithidia* incidence? Selective disappearance may resolve this apparent contradiction. The queens that emerge in spring are a small subset of the whole, and the high‐quality queens who survived particularly harsh winters may differ from the wider population. *Crithidia* can exacerbate weight loss in hibernating queens (Brown et al., 2003), and because body mass is an important predictor of overwinter survival (Beekman et al., 1998), small infected queens may not tolerate this high metabolic cost or survive to feature in these data. Similarly, if warm winters elevate overwinter mortality, particularly in low‐quality queens, then the high‐quality survivors that feature in this dataset may survive well subsequently. These ideas need testing.

While these results offer insight into how climatic variation affects wild bees, there is more to be done. We analysed field‐caught queens that were brought into the lab and then buffered from variation in climate and resource availability. This is not too dissimilar from the field: once queens have produced workers, they remain inside the nest where they are provisioned by workers who also regulate nest temperatures (Gradišek et al., 2023). However, this thermal buffering is not absolute, and as global temperatures rise further, nests may act as heat traps that amplify the impacts of warming (Woods et al., 2021). Additionally, we study bees in the middle of their range, and our analyses do not capture the impact of extreme weather events (heatwaves, drought, flooding) that are rising in frequency (Easterling et al., 2000) and could be as detrimental (or more so) than shifts in mean climatic conditions. That is to say, the impacts of climate that we describe here are likely modest compared to those experienced in the field. Future work across the species' range, linking climatic variation to the production of queens and drones, could offer powerful insights into how climate affects wild bees. Finally, our results hint at the potential for parasitism to influence how different populations respond to climatic variation; for example, study site variation in *Crithidia* incidence could contribute towards site variation in mass, and in turn, survival and reproduction. Testing the role of parasites in mediating host responses to climatic change represents an interesting avenue of investigation.

If it is the case that climatic factors that hinder resource acquisition in summer, and exacerbate resource loss overwinter, are particularly detrimental, then climate change and forage quality likely interact to affect bumblebee queen fitness. This is not surprising given that nutrition is a central determinant of bumblebee fitness (Archer et al., 2021; Stabler et al., 2015; Vaudo et al., 2016). This result also highlights that climate and land‐use intensification may act in synergy, as land‐use and resource acquisition in bumblebees are linked (Birkenbach et al., 2025; Straub et al., 2023, 2025). This suggests a mitigation strategy; while climatic change can only be addressed by global efforts, providing more floral resources during crucial windows of colony development may buffer bees from its detrimental impacts. This could be achieved by optimising seed mixes that are already deployed in schemes aimed at improving pollinator health in agricultural environments, that is by including late‐blooming flowers (Nichols et al., 2022). Given that some insects can rapidly respond to local habitat improvements (Blüthgen et al., 2023; Doublet et al., 2022; Halsch et al., 2021), this offers the hope that improving bumblebee queen nutrition at key time points could somewhat mitigate the deleterious effects of climatic change.

## AUTHOR CONTRIBUTIONS

C. Ruth Archer performed analyses and wrote the first draft. Lena Wilfert and Regula Schmid‐Hempel collected data. Paul Schmid‐Hempel, Lena Wilfert and Regula Schmid‐Hempel edited the manuscript. All authors helped conceive the manuscript.

## CONFLICT OF INTEREST STATEMENT

None to declare.

## Supporting information


**Table S1.** Location information for each study site and weather station that provide climatic data.
**Table S2.** Trait specific sample sizes.
**Table S3.** A summary of the baseline models used in the sliding window analyses.
**Table S4.** Dates that each window in sliding window analyses corresponds.
**Table S5.** Results of moving window analyses for (A) precipitation and (B) temperature data.
**Figure S1.** Map of Switzerland showing the location of study sites and associated weather stations.
**Figure S2.** Variation in queen collection dates in each year of sampling at Neunforn (blue triangles) and Aesch (red circles).
**Figure S3.** Mean daily air temperature in July (A, B), annual temperature norms (C, D) and annual precipitation norms (E, F) across Switzerland in 1961–1990 (A, C, E) and 1981–2010 (B, D, F) (*Source:* www.geo.admin.ch).
**Figure S4.** Annual temperature (A) and precipitation (B) recorded at Neunforn (blue triangles) and Aesch (red circles) from 1999 to 2013.
**Figure S5.** The correlation between annual temperature and precipitation at Neunforn (blue) and Aesch (red) from 1999 to 2013.
**Figure S6.** Starting model for the piecewise SEM analyses. This schematic illustrates the full model used in the piecewise SEM process.
**Figure S7.** The predicted relationship between *Crithidia* infection and queen body mass (g), collection day of the year (DOY) and standardised temperature (°C).
**Figure S8.** Schematic summary of sliding window analyses.
**Figure S9.** Sliding window analyses results summary—body mass.
**Figure S10.** Sliding window analyses results summary—*Crithidia*.
**Figure S11.** Sliding window analyses results summary—survival.
**Figure S12.** Sliding window analyses results summary—reproduction, that is was a colony produced/not, as signified by worker production.

## Data Availability

Data available from the FigShare digital repository: 10.6084/m9.figshare.30069484 (Archer et al., 2025).
