# Supplementary material for: Complex effects of climatic variation on bumblebee queen fitness
Source: J Anim Ecol. 2025 Sep 19;94(12):2448–60. doi: 10.1111/1365-2656.70140 (PMC12673246; doi:10.1111/1365-2656.70140)
Supplement: Supplementary file 1 — Table S1. Location information for each study site and weather station that provide climatic data. Table S2. Trait specific sample sizes. Table S3. A summary of the baseline models used in the sliding window analyses. Table S4. Dates that each window in sliding window analyses corresponds. Table S5. Results of moving window analyses for (A) precipitation and (B) temperature data. Figure S1. Map of Switzerland showing the location of study sites and associated weather stations. Figure S2. Variation in queen collection dates in each year of sampling at Neunforn (blue triangles) and Aesch (red circles). Figure S3. Mean daily air temperature in July (A, B), annual temperature norms (C, D) and annual precipitation norms (E, F) across Switzerland in 1961–1990 (A, C, E) and 1981–2010 (B, D, F) (Source: www.geo.admin.ch). Figure S4. Annual temperature (A) and precipitation (B) recorded at Neunforn (blue triangles) and Aesch (red circles) from 1999 to 2013. Figure S5. The correlation between annual temperature and precipitation at Neunforn (blue) and Aesch (red) from 1999 to 2013. Figure S6. Starting model for the piecewise SEM analyses. This schematic illustrates the full model used in the piecewise SEM process. Figure S7. The predicted relationship between Crithidia infection and queen body mass (g), collection day of the year (DOY) and standardised temperature (°C). Figure S8. Schematic summary of sliding window analyses. Figure S9. Sliding window analyses results summary—body mass. Figure S10. Sliding window analyses results summary—Crithidia. Figure S11. Sliding window analyses results summary—survival. Figure S12. Sliding window analyses results summary—reproduction, that is was a colony produced/not, as signified by worker production. [file JANE-94-2448-s001.docx]

**COMEPLEX EFFECTS OF CLIMATIC VARIATION ON BUMBLEBEE QUEEN FITNESS**

**CONTENTS**

| PART 1 – STUDY SITE LOCATION | Page 2 |
| --- | --- |
| PART 2 – COLLECTION DATES AND SAMPLE SIZES | Page 4 |
| PART 3 – SITE CLIMATIC INFORMATION | Page 6 |
| PART 4 – EXTRA DETAIL STATISTICS | Page 11 |
| PART 5 – EXTRA INFORMATION ANNUAL RESULTS | Page 14 |
| PART 6 – EXTRA INFORMATION SLIDING WINDOWS | Page 15 |

**PART 1 – STUDY SITE LOCATION**

**Fig S1. Map of Switzerland showing the location of study sites and associated weather stations.** Circles show the study sites (western sites in red – Aesch, eastern sites in blue – Neunforn), while squares show the associated weather stations. For Neunforn, weather data originated from two sites: precipitation data were provided by the nearby Niederneunforn station, while temperature data originated from the Aadorf / Tänikon weather station (south-east of the study site).


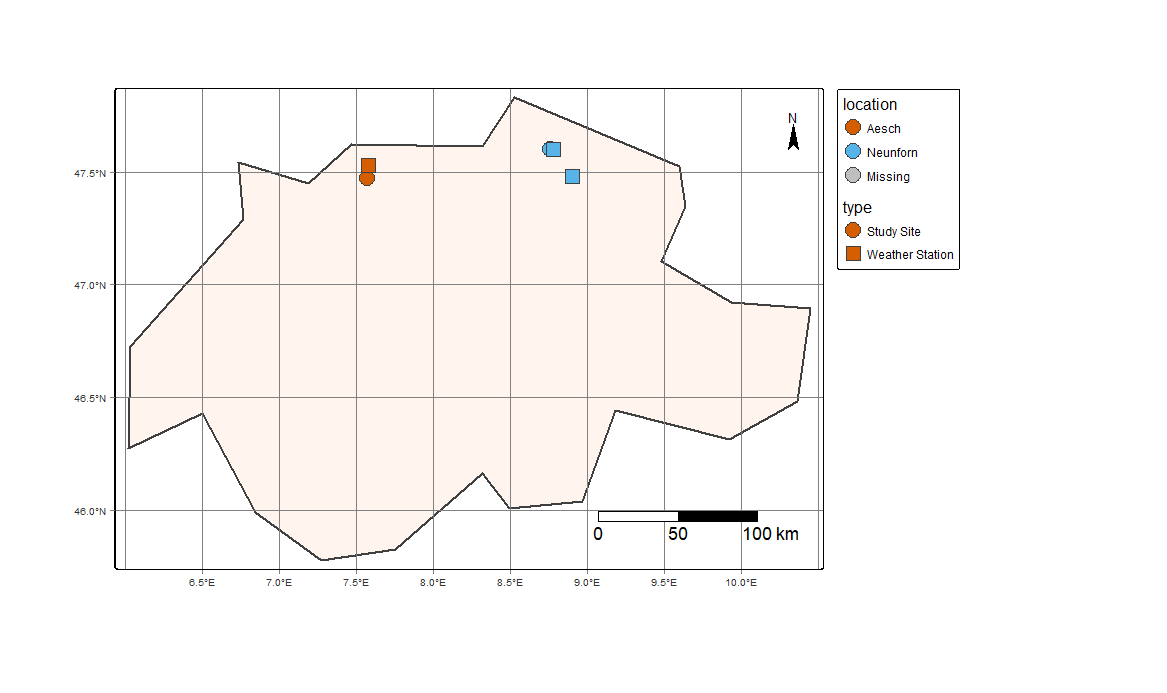


**Table S1. Location information for each study site and weather station that provide climatic data.** Geographic information for each weather station are exact and provided by MeteoSchweiz, while geographic data for study sites correspond to the approximate location where bees were collected.

| Location | Description | Elevation (meters) | Longitude / Latitude |
| --- | --- | --- | --- |
| Aesch | Study Site | 309 | 7° 34′ / 47° 28′ |
| Basel / Binningen | Weather station | 316 | 7°35' / 47°32' |
| Neunforn | Study Site | 460 | 8° 46′ / 47° 36′ |
| Aadorf / Tänikon | Weather station | 539 | 8°54' / 47°29' |
| Niederneunforn | Weather station | 440 | 8°47' / 47°36' |

**PART 2 – COLLECTION DATES AND SAMPLE SIZES**

**Fig S2. Variation in queen collection dates in each year of sampling at Neunforn (blue triangles) and Aesch (red circles).** The date of the year (1 = January first) when each queen in the data-set was collected, presented by year. The 1^st^ of March is the reference date for sliding window analyses, and is shown as an inverted triangle on the x-axis.


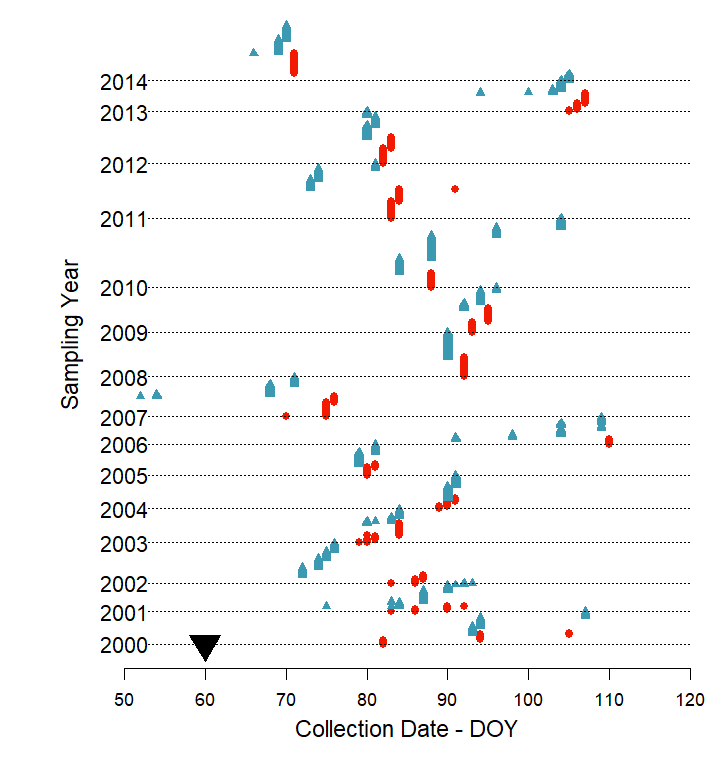


**Table S2. Trait Specific Sample Sizes**. The total column refers to the number of queens collected. However, because not all traits were measured in all animals, sample sizes differ between the variables of interest. The numbers below reflect the number of “complete cases” that were used in the model for each trait i.e. observations where data were available for the response variable of interest and relevant covariates (mass, collection date). SEM stands for the piecewiseSEM analyses. In 2012 and 2013 mass was not recorded and because this is a vital co-variate that includes expression of all other traits assayed, queens lacking mass information were not included in analyses.

| Year | Total | Crithidia | Mass | Survival | Reproduction | SEM |
| --- | --- | --- | --- | --- | --- | --- |
| 2000 | 243 | 243 | 243 | 130 | 242 | 242 |
| 2001 | 212 | 203 | 209 | 67 | 167 | 162 |
| 2002 | 304 | 248 | 303 | 133 | 139 | 116 |
| 2003 | 254 | 244 | 247 | 230 | 235 | 232 |
| 2004 | 253 | 250 | 253 | 249 | 253 | 250 |
| 2005 | 233 | 214 | 232 | 180 | 180 | 166 |
| 2006 | 205 | 187 | 196 | 187 | 188 | 179 |
| 2007 | 300 | 294 | 299 | 205 | 282 | 277 |
| 2008 | 335 | 326 | 334 | 289 | 292 | 285 |
| 2009 | 337 | 312 | 336 | 314 | 314 | 290 |
| 2010 | 518 | 501 | 518 | 234 | 235 | 231 |
| 2011 | 409 | 379 | 406 | 333 | 333 | 316 |
| 2012 | 390 | NA | NA | NA | NA | NA |
| 2013 | 283 | NA | NA | NA | NA | NA |
| 2014 | 364 | 349 | 360 | 352 | 353 | 343 |
| Total | 4640 | 3750 | 3936 | 2903 | 3213 | 3089 |

**PART 3 – SITE CLIMATIC INFORMATION**

**Fig S3. Mean daily air temperature in July (A, B), annual temperature norms (C,D) and annual precipitation norms (E, F) across Switzerland in 1961-1990 (A, C, E) and 1981-2010 (B, D, F) (source:** [**www.geo.admin.ch**](http://www.geo.admin.ch)**).** Maps were created by the Federal Office for the Environment (FOEN) and were accessed on the 13.10.23 online at the portal [www.geo.admin.ch](http://www.geo.admin.ch). The legends show how colours correspond to (in each panel from left to right) temperature in graphs A and B, temperature in graphs C and D and rainfall in graphs E and F. The full gradient of colours used in each map is not shown here, but an evenly spaced snapshot of colours across the full gradient is shown. In each graph, the locations of study locations are marked – although these cannot be seen readily in panels B-F.

**
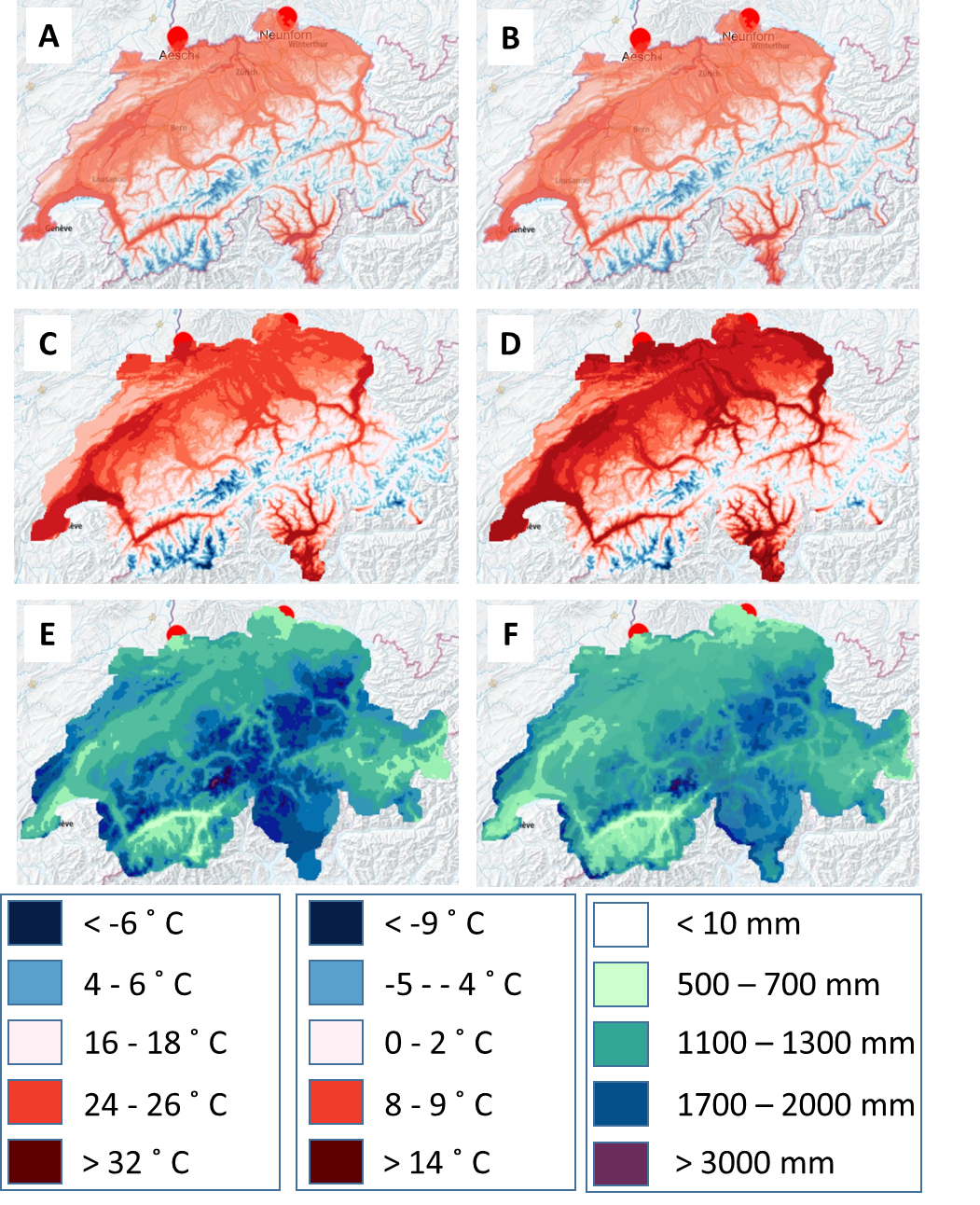
**

**Fig S4. Annual temperature (A) and precipitation (B) recorded at Neunforn (blue triangles) and Aesch (red circles) from 1999-2013.** Temperature measures reflect air temperature 2 m above ground reported as deviation of the annual mean to the norm – the norm being the average air temperature 2m above the ground between 1991 and 2020. Precipitation measures include snowfall, rain and hail and are reported as percentages, and reflect the relation of the annual total to the norm in the period 1991-2020. The dashed lined in each graph shows where points would sit if there were no deviation from the historic norm.

**
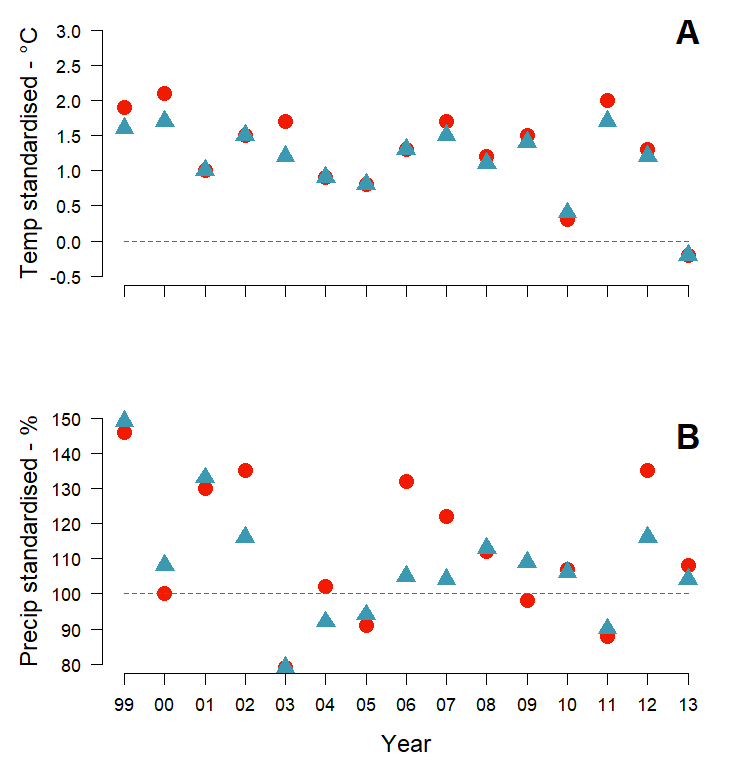
**

**Fig S5. The correlation between annual temperature and precipitation at Neunforn (blue) and Aesch (red) from 1999-2013.** Regression lines were created using *abline* and show the correlation between climatic variables at each site.


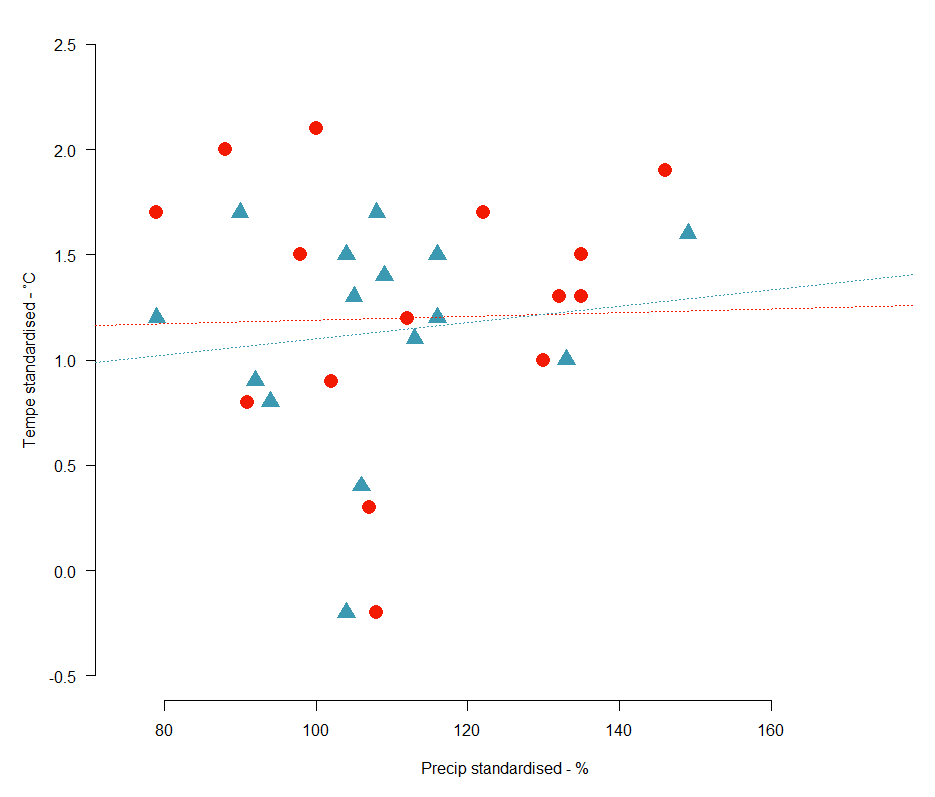


**PART 4 – EXTRA DETAIL STATISTICS**

**Text S1 – Survival Model**

We analyzed survival data in a time-to-event framework, where the event was dying within the observation period (dead = 1, alive = 0) and a “time” is associated with each outcome. For animals that died, this “time” is the approximate age at death (i.e. date of death minus the date each queen was collected from the field). For bees that survived, this age was when bees were recorded as being alive for the last time. These final observations may have been made when noting when queens were transferred to a new box, laid eggs, produced workers or were frozen (e.g. date of egg laying minus the date each queen was collected from the field). Because survival was not monitored at a regular schedule after around mid-June when colonies were established for experiments, all observations are capped on the 166^th^ day of the year. Any bees still alive on this date are recorded as being alive and given a censoring time equivalent to their approximate age on the 166^th^ day of the year (e.g. 166 minus the day of the year each queen was collected from the field). Any survival observations that could not be associated with a date were excluded from analyses. The model structure for survival analyses was otherwise identical to those described for other traits.

**Figure S6. Starting model for the piecewise SEM analyses.** This schematic illustrates the full model used in the piecewise SEM process. Each arrow demonstrates a hypothesis i.e. a possible relationship between the variables studied and the direction of effect. A) This is the full model, where climatic variables are presented in dark blue and hypothetical climatic effects shown as solid dark blue arrows. Variables associated with where (site) or when (collected day of the year (DOY)) queens were collected are shown in green and associated arrows are shown with narrow dashes. Relationships between response variables are shown as wide dashed arrows linking body mass (purple) with *Crithidia* (grey) and colony establishment (i.e. reproduction – yellow). For clarity, panel B shows only arrows involving climatic variables, panel C shows only collection site / date information and panel D, relationships between response traits. This separation is just to allow Readers to clearly see the full model design; all of these relationships were included in a single model.

**
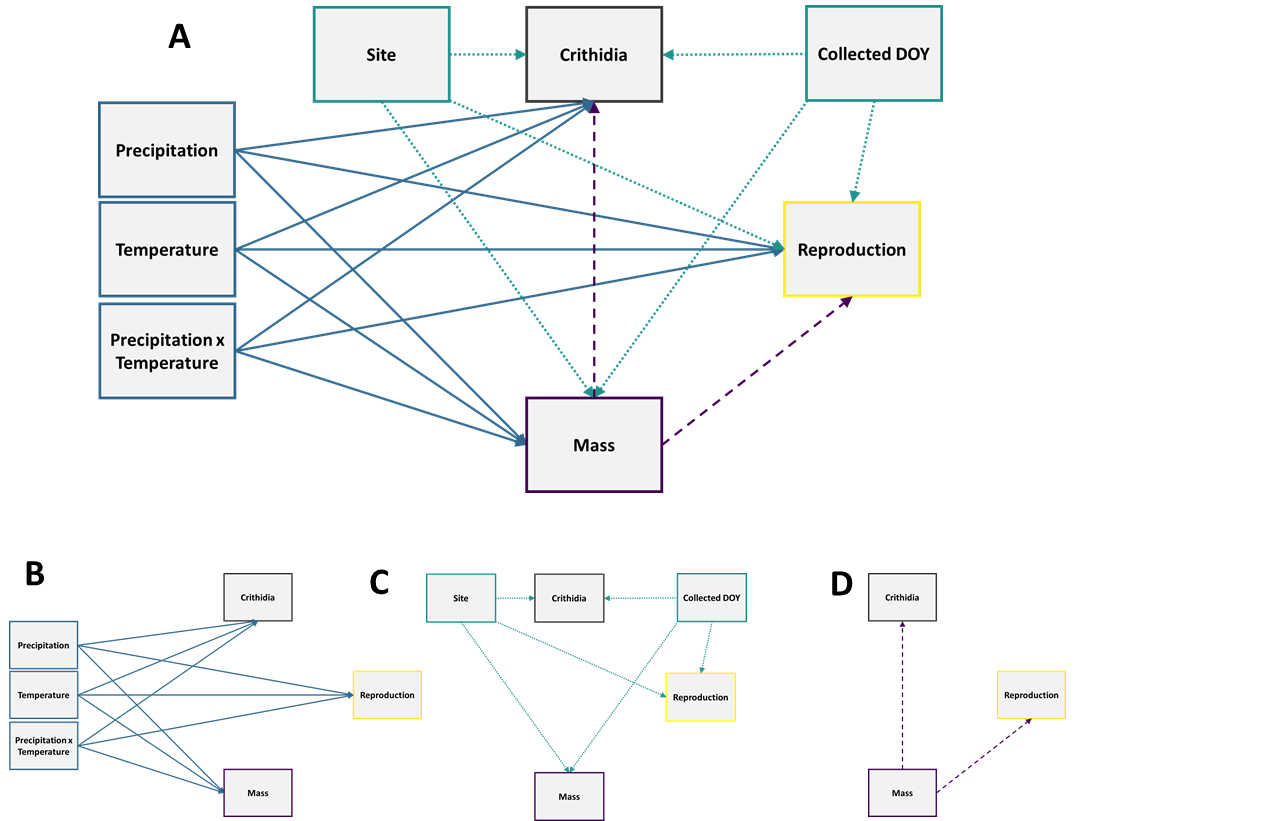
**

**PART 5 – EXTRA INFORMATION ANNUAL RESULTS**

**Figure S7. The predicted relationship between *Crithidia* infection and queen body mass (g), collection day of the year (DOY) and standardised temperature (ᵒC).** Shaded regions show confidence intervals, and lines show predictions from the preferred model estimated using the *ggpredict* function in the *ggeffects* package. Red = site Aesch, Blue = site Neunforn.

**
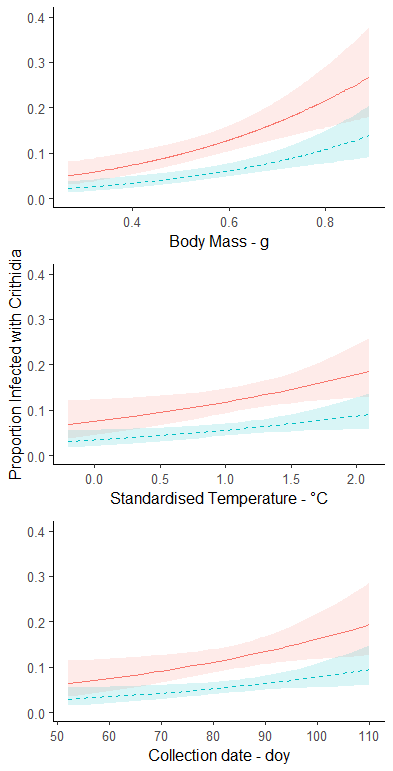
**

**PART 6 – EXTRA INFORMATION SLIDING WINDOWS**

**Figure S8. Schematic summary of sliding window analyses.** Here, the starting date that is used as a reference is shown by a red dashed line and arrow. A climate variable (e.g. rainfall) in every possible time-step (e.g. here, a day) in the predefined time-window (here, the preceding 6 days) is related the trait of interest (e.g. bee reproduction) in a single model. These time steps are shown by the blue lines. Here, climate in 21 windows is related to bee reproduction in a separate model. These models are then compared to see which provides the strongest fit to the data – the direction and magnitude of effect can be extracted from this single model. If multiple models provide a strong fit, model averaging is used to extract climatic effects on the trait of interest. Randomisation testing is used to test that the result obtained is unlikely to have arisen by chance alone, given multiple testing. Figure created in BioRender.

**
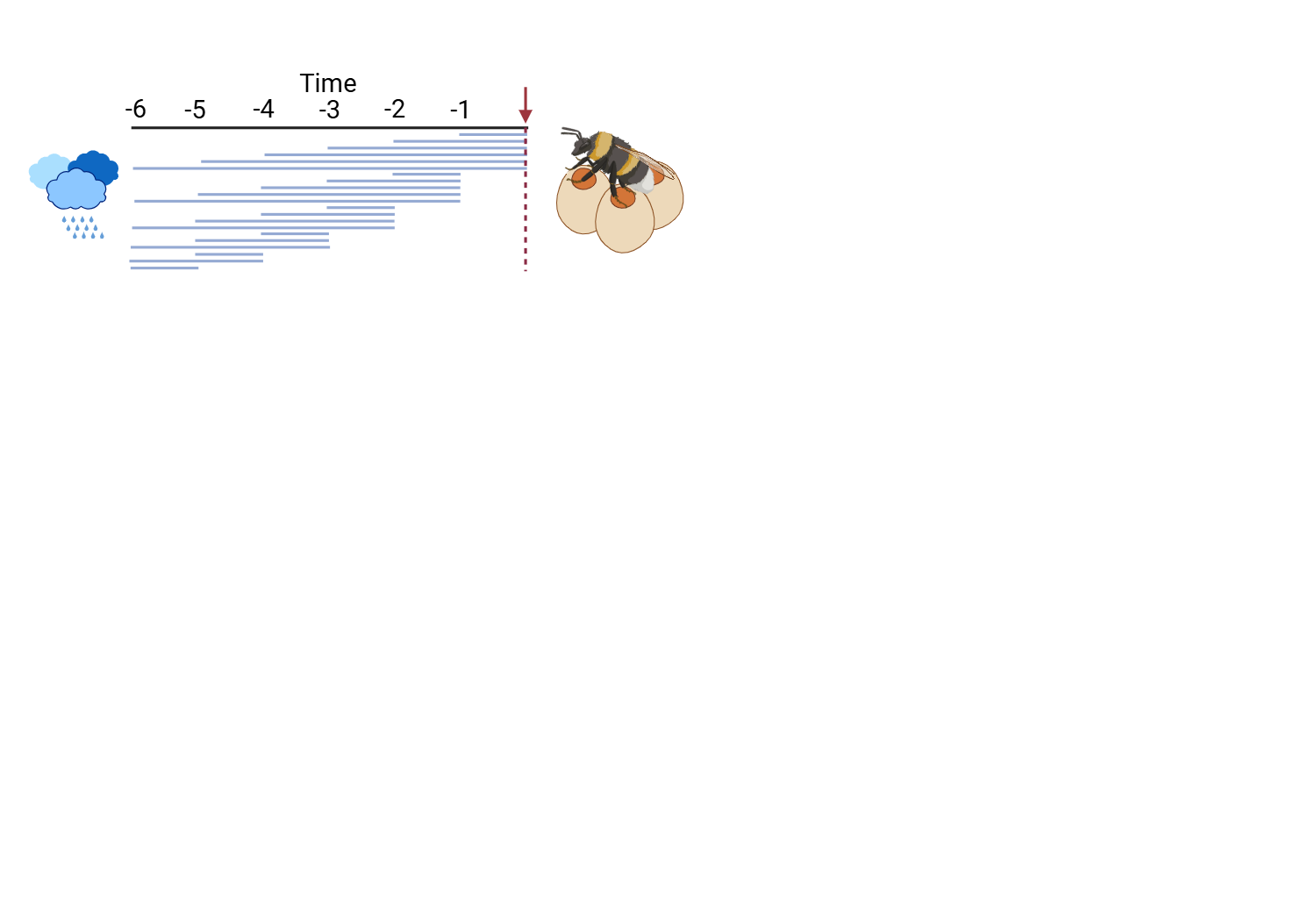
**

**Table S3. A summary of the baseline models used in the sliding window analyses.** Baseline models exclude the effects of climate – these are modelled in different time windows as described above. While we attempted to include year as a random effect throughout, we could not do this due to convergence issues.

| **Response** | **Baseline Model** |
| --- | --- |
| **Mass** | lmer(Mass ~ scale(collected_doy)+(1\|Year)) |
| **Crithidia** | glm(Crithidia ~ scale(Mass)+scale(collected_doy)+Year, family="binomial") |
| **Survival** | coxph(Surv(survival_object) ~ scale(Mass)+scale(collected_doy)+Year) |
| **Reproduction** | glm(reproduction ~ scale(Mass)+scale(collected_doy)+Year, family="binomial" |

**Figure S9-12. Output of initial sliding window analyses.** Results are shown separately for each trait but all figures are described here. For each trait, there are four graphs – two showing the results for temperature and two for precipitation. For each climatic variable / trait combination, the “model fit” graph shows the difference in AIC values between a model where a window opens in each time step, and closes in each time step, relative to a model that excludes climate. The greater divergence between these values, the better the fit. Colour key (with ∆AIC values) is indicated in the graph: red values show large ∆AIC values, showing that a model performs well compared to the baseline null model, while purple values show a minimal difference between the baseline null model and climate model. The graphs describing “effect” summarise “beta” and show the relationship between climate and trait at each point. The resolution of the graphs for temperature and precipitation differ – results for temperature show weekly steps, precipitation monthly steps. Window 0 = 1^st^ March in year queens collected.

**Fig S9. Sliding Window Analyses Results Summary – Body Mass**

**
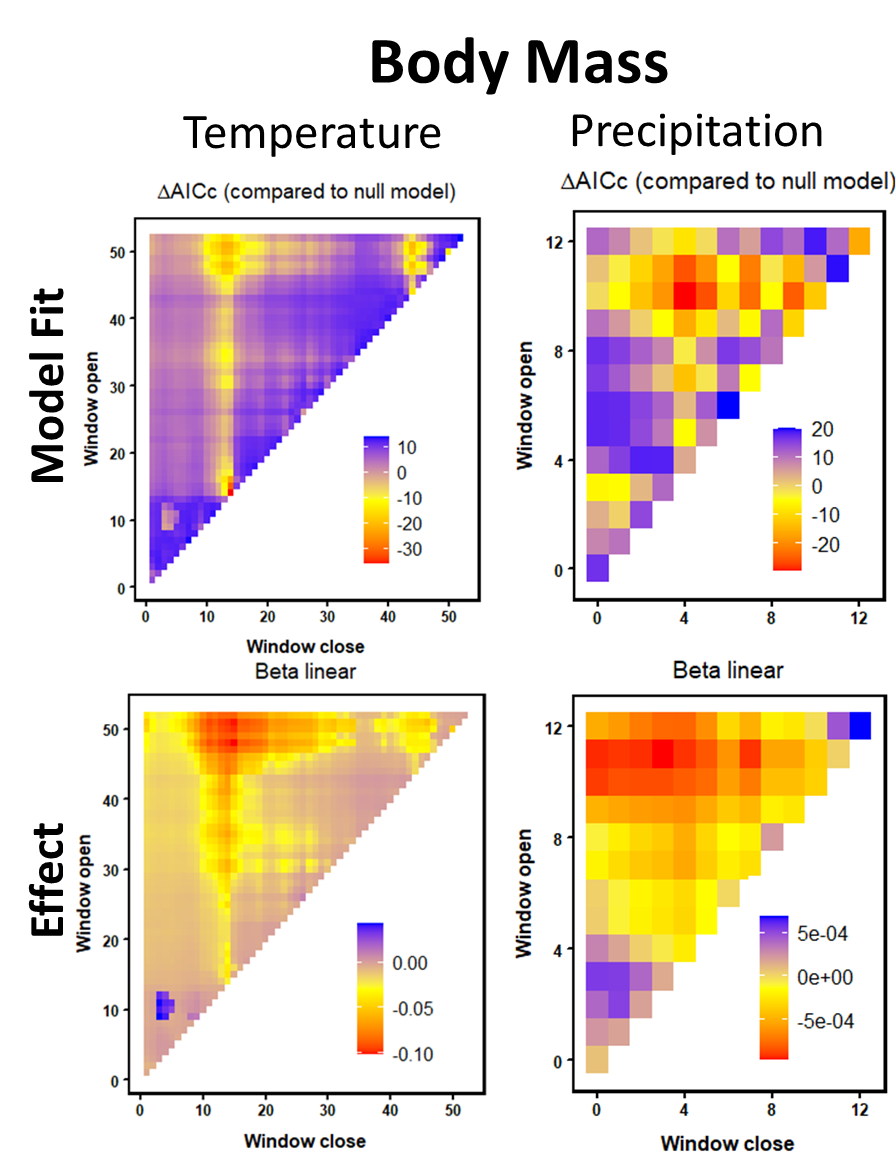
**

**Fig S10. Sliding Window Analyses Results Summary – *Crithidia***

**
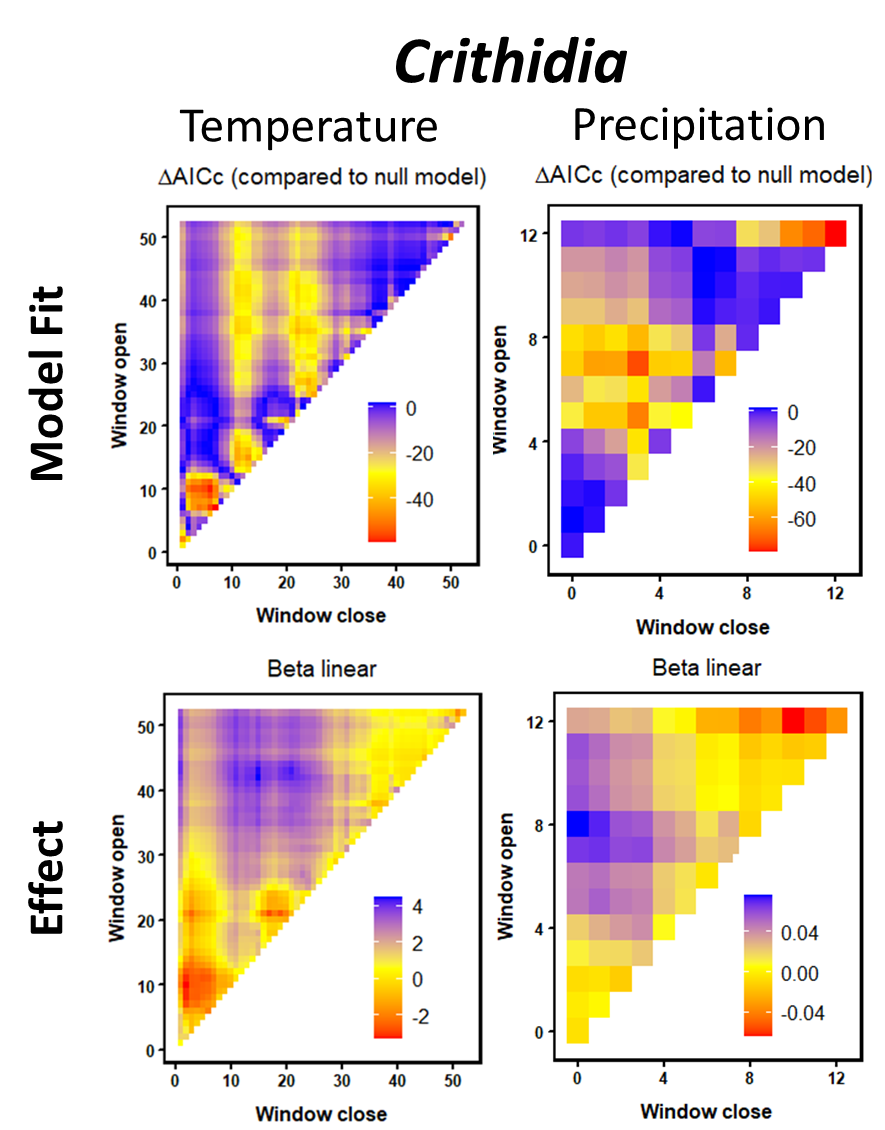
**

**Fig S11. Sliding Window Analyses Results Summary – Survival**

**
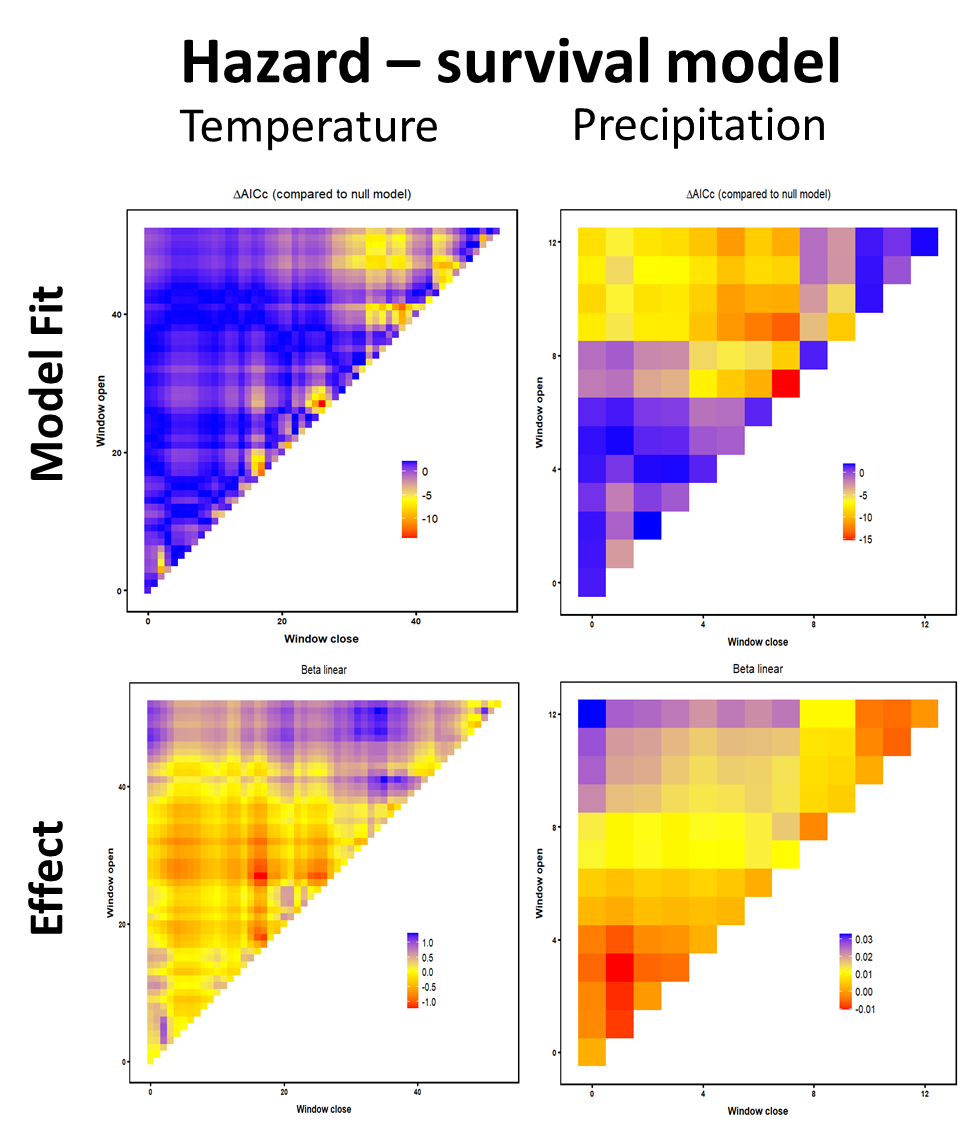
**

**Fig S12. Sliding Window Analyses Results Summary – Reproduction i.e. was a colony produced / not, as signified by worker production.**

**
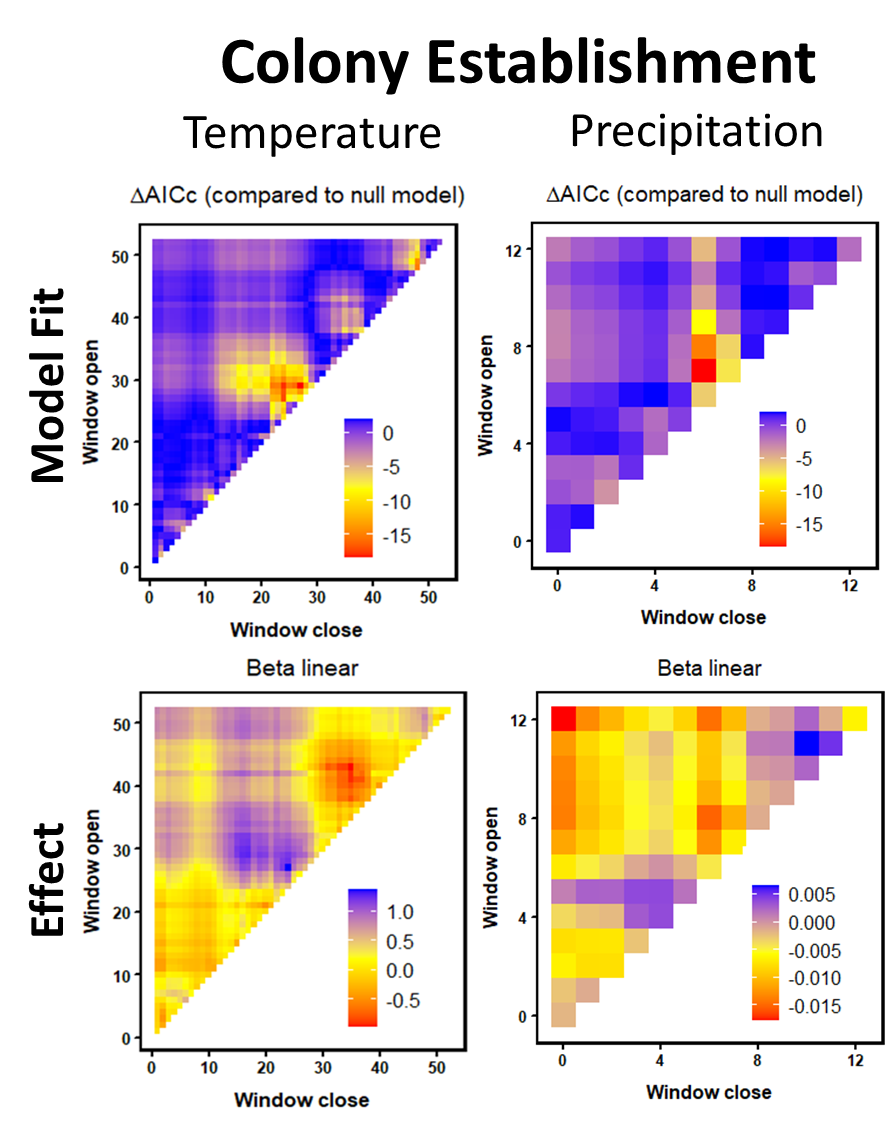
**

**Table S4. Dates that each window in sliding window analyses corresponds.** These dates are for a non-leap year but are intended to help interpret the figures shown above.

| **Window – Temp** | **Corresponding Date** | **Window - Precipitation** |
| --- | --- | --- |
| 0 | 1^st^ March | 0 |
| 1 | 22^nd^ Feb | 1 |
| 2 | 15^th^ Feb |  |
| 3 | 8^th^ Feb |  |
| 4 | 1^st^ Feb |  |
| 5 | 25^th^ Jan | 2 |
| 6 | 18^th^ Jan |  |
| 7 | 11^th^ Jan |  |
| 8 | 4^th^ Jan |  |
| 9 | 28^th^ Dec | 3 |
| 10 | 21^st^ Dec |  |
| 11 | 14^th^ Dec |  |
| 12 | 7^th^ Dec |  |
| 13 | 30^th^ Nov | 4 |
| 14 | 23^rd^ Nov |  |
| 15 | 16^th^ Nov |  |
| 16 | 9^th^ Nov |  |
| 17 | 2^nd^ Nov |  |
| 18 | 26^th^ Oct | 5 |
| 19 | 19^th^ Oct |  |
| 20 | 12^th^ Oct |  |
| 21 | 5^th^ Oct |  |
| 22 | 28^th^ Sept | 6 |
| 23 | 21^st^ Sept |  |
| 24 | 14^th^ Sept |  |
| 25 | 7^th^ Sept |  |
| 26 | 31^st^ Aug | 7 |
| 27 | 24^th^ Aug |  |
| 28 | 17^th^ Aug |  |
| 29 | 10^th^ Aug |  |
| 30 | 3^rd^ Aug |  |
| 31 | 27^th^ July | 8 |
| 32 | 20^th^ July |  |
| 33 | 13^th^ July |  |
| 34 | 6^th^ July |  |
| 35 | 29^th^ June | 9 |
| 36 | 22^nd^ June |  |
| 37 | 15^th^ June |  |
| 38 | 8^th^ June |  |
| 39 | 1^st^ June |  |
| 40 | 25^th^ May | 10 |
| 41 | 18^th^ May |  |
| 42 | 11^th^ May |  |
| 43 | 4^th^ May |  |
| 44 | 27^th^ April | 11 |
| 45 | 20^th^ April |  |
| 46 | 13^th^ April |  |
| 47 | 6^th^ April |  |
| 48 | 30^th^ March | 12 |
| 49 | 23^rd^ March |  |
| 50 | 16^th^ March |  |
| 51 | 9^th^ March |  |
| 52 | 2^nd^ March |  |

**Table S5. Results of moving window analyses for (A) Precipitation and (B) Temperature data.** The best performing models always included climate as a linear (rather than a quadratic) term.

| **Model** | **Window** | | **∆AIC** | ***ß*** | **SE** | ***Wi*** | ***P ΔAICc*** | ***P*_C_** |
| --- | --- | --- | --- | --- | --- | --- | --- | --- |
|  | **Start** | **End** |  |  |  |  |  |  |
| 1. ***Precipitation*** |  |  |  |  |  |  |  |  |
| ***Body Mass (g)*** |  |  |  |  |  |  |  |  |
| *Best Model*  *Confidence Set* | 10  (10) | 4  (4.5) | -29.45 | -0.001  (-0.001) | 0.003 | 0.674 | **< 0.001** | **0.008** |
| ***Crithidia* (0 / 1)** |  |  |  |  |  |  |  |  |
| *Best Model* | 12 | 12 | -78.94 | -0.034 | 0.058 | 0.926 | **< 0.001** | **< 0.001** |
| **Reproduction (0 / 1)** |  |  |  |  |  |  |  |  |
| *Best Model* | 7 | 6 | -18.46 | -0.013 | 0.038 | 0.826 | **< 0.001** | **< 0.001** |
| **Mortality risk** |  |  |  |  |  |  |  |  |
| *Best Model*  *Confidence Set* | 7  (10) | 7  (5) | -15.23 | 0.011  (0.014) | 0.003 | 0.305 | **< 0.001** | **0.009** |
| 1. ***Temperature*** |  |  |  |  |  |  |  |  |
| **Mass (g)** |  |  |  |  |  |  |  |  |
| *Best Model* | 14 | 14 | -35.87 | -0.025 | 0.003 | 0.920 | **< 0.001** | **0.003** |
| ***Crithidia*** |  |  |  |  |  |  |  |  |
| *Best Model*  *Confidence Set* | 7  (10) | 7  (6) | -59.18 | -1.237  (-1.709) | 0.060 | 0.377 | **< 0.001** | **0.001** |
| **Reproduction (0 / 1)** |  |  |  |  |  |  |  |  |
| *Best Model*  *Confidence Set* | 29  (30) | 27 (23) | -18.41 | 0.972  (1.020) | 0.039 | 0.174 | **< 0.001** | **0.001** |
| ***Mortality risk*** |  |  |  |  |  |  |  |  |
| Range – all year |  |  |  |  |  |  |  |  |
| *Best Model* | 27  (44) | 26  (23) | -14.14 | -1.008  (0.176) | 0.257 | 0.182 |  |  |
| Range 0-19 |  |  |  |  |  |  |  |  |
| *Best Model*  *Confidence Set* | 17  (12) | 17  (5.5) | -11.70 | -0.724  (-0.493) | 0.201 | 0.371 | **< 0.001** | **0.027** |
| Range 20-29 |  |  |  |  |  |  |  |  |
| *Best Model*  *Confidence Set* | 27  (27) | 26  (25.5) | -14.14 | -1.008  (-0.841) | 0.257 | 0.706 | **< 0.001** | **0.002** |
| Range 30-52 |  |  |  |  |  |  |  |  |
| *Best Model*  *Confidence Set* | 41  (47) | 38  (36) | -12.26 | 1.072  (0.883) | 0.285 | 0.142 | **0.002** | 0.053 |

Windows for precipitation data are measured in months, while windows for temperature data are measured in weeks. For traits where there was one clear climate peak, and the best model from the sliding window analyses had a high probability of being the best model in the model set, we only report parameters associated with the best model. For traits where there was one clear climate peak, but multiple models have a similar probability of being the best model in the model set, we also report a *ß* value based on model averaging on the confidence set – a subset of models where we can be 95% confident that the best model is included – and median windows for the set. These values are provided in parenthesis. For the models relating climate to survival, three distinct peaks were suggested by the overall analyses. Thus, individual moving windows were run for periods corresponding to each peak (0 – 19 weeks prior to 1.3; 20 – 29 weeks, 30 – 52 weeks). The confidence set was calculated for each of these ranges and significance testing carried out for each peak. **∆AIC** - the AIC value of the model reported, subtracted from the AIC of the baseline model (i.e. a model including climate only). These values are comparable across traits – more negative values indicate a stronger influence of the climatic variable tested in that model. ***ß*** *–* relationship between the climatic variable tested and the response variable. For example, the value of -0.001 *ß* for the model linking body mass and precipitation indicates that for each increase in precipitation (% relative to historical norm), body mass declines by -0.001g. Note that because survival data were analysed in a cox model framework, here beta reflects the reduction in hazard i.e. risk of dying. **SE** – standard error around the estimate of *ß.* ***Wi –*** the probability that the model reported, is the best model within the model set. ***PΔAICc, P*_C_ *–*** results of randomisation analyses, the likelihood of obtaining the level of model support observed for the best model by chance. ***PΔAICc*** is the more accurate metric when running >100 iterations (i.e. as here). ***P*_C_** is more accurate when fewer iterations are run due to limitations with computation power. Here, P values were always calculated with 500 iterations and degrees of freedom calculated as the number of years of data included in the analyses, multiplied by the number of sites.
